# Proportional assist ventilation versus pressure support ventilation for weaning from mechanical ventilation in adults: a meta-analysis and trial sequential analysis

**DOI:** 10.1186/s13054-020-03251-4

**Published:** 2020-09-14

**Authors:** Liang-Jun Ou-Yang, Po-Huang Chen, Hong-Jie Jhou, Vincent Yi-Fong Su, Cho-Hao Lee

**Affiliations:** 1 Department of Physical Medicine and Rehabilitation, Chang Gung Memorial Hospital, Linkou, Taoyuan, Taiwan, Republic of China; 2 Department of Internal Medicine, Tri-Service General Hospital, National Defense Medical Center, Taipei, Taiwan, Republic of China; 3 Department of General Medicine, Tri-Service General Hospital, National Defense Medical Center, Taipei, Taiwan, Republic of China; 4 Department of Neurology, Changhua Christian Hospital, Changhua, Taiwan, Republic of China; 5 Department of Internal Medicine, Taipei City Hospital, Taipei City Government, Taipei, Taiwan, Republic of China; 6 Department of Chest Medicine, Taipei Veterans General Hospital, Taipei, Taiwan, Republic of China; 7 Faculty of Medicine, School of Medicine, National Yang-Ming University, Taipei, Taiwan, Republic of China; 8 Division of Hematology and Oncology Medicine, Department of Internal Medicine, Tri-Service General Hospital, National Defense Medical Center, Taipei, Taiwan, Republic of China

**Keywords:** Proportional assist ventilation, Pressure support ventilation, Ventilator weaning, Systemic review, Meta-analysis, Trial sequential analysis

## Abstract

**Background:**

Pressure support ventilation (PSV) is the prevalent weaning method. Proportional assist ventilation (PAV) is an assisted ventilation mode, which is recently being applied to wean the patients from mechanical ventilation. Whether PAV or PSV is superior for weaning remains unclear.

**Methods:**

Eligible randomized controlled trials published before April 2020 were retrieved from databases. We calculated the risk ratio (RR) and mean difference (MD) with 95% confidence intervals (CIs).

**Results:**

Seven articles, involving 634 patients, met the selection criteria. Compared to PSV, PAV was associated with a significantly higher rate of weaning success (fixed-effect RR 1.16; 95% CI 1.07–1.26; *I*^2^ = 0.0%; trial sequential analysis-adjusted CI 1.03–1.30), and the trial sequential monitoring boundary for benefit was crossed. Compared to PSV, PAV was associated with a lower proportion of patients requiring reintubation (RR 0.49; 95% CI 0.28–0.87; *I*^2^ = 0%), a shorter ICU length of stay (MD − 1.58 (days), 95% CI − 2.68 to − 0.47; *I*^2^ = 0%), and a shorter mechanical ventilation duration (MD − 40.26 (hours); 95% CI − 66.67 to − 13.84; *I*^2^ = 0%). There was no significant difference between PAV and PSV with regard to mortality (RR 0.66; 95% CI 0.42–1.06; *I*^2^ = 0%) or weaning duration (MD − 0.01 (hours); 95% CI − 1.30–1.28; *I*^2^ = 0%).

**Conclusion:**

The results of the meta-analysis suggest that PAV is superior to PSV in terms of weaning success, and the statistical power is confirmed using trial sequential analysis.

**Graphical abstract:**

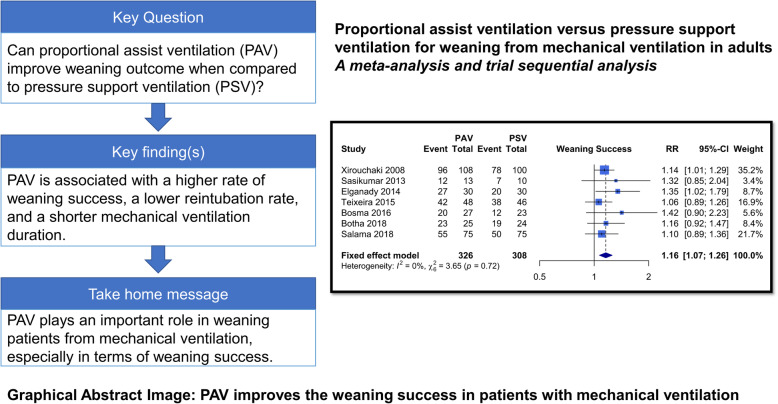

## Background

Acute respiratory failure and intubation is a common scenario in critically ill patients. After intubation, the primary treatment goal is liberation from the ventilator, restoration of the patient’s ability to breathe independently and, further, removal of the endotracheal tube. The spontaneous breathing trial (SBT), which assesses the patient’s ability to breathe while receiving minimal or no ventilator support, is used to wean patients from mechanical ventilation. Common methods include the T-piece, which involves an immediate shift from full ventilator support to a period of breathing without ventilator assistance, and a program of pressure support ventilation (PSV), which gradually reduces the amount of ventilator support. Delayed weaning may lead to complications, such as ventilator-associated pneumonia, ventilator-induced lung injury, or ventilator-induced diaphragmatic dysfunction [1]. However, premature extubation may cause reintubation, aspiration, or respiratory muscle fatigue, leading to a higher rate of morbidity and mortality [2]. Defining suitable candidates for weaning is of critical importance in clinical practice.

Subira et al. stated that, compared to 2 h of T-piece ventilation, 30 min of PSV led to significantly higher successful extubation rates [3]. However, patient-ventilator asynchrony is a burden for patients with PSV. The asynchrony may be caused by factors related to the patient, the ventilator, or both, including patient characteristics, depth of sedation, ventilator settings, and operational principles [4]. Patient-ventilator asynchrony [5] increases the patient’s work of breathing, resulting in respiratory muscle fatigue. It worsens the extent of respiratory failure, prolonging the weaning time and length of intensive care unit (ICU) stay [6].

Proportional assist ventilation (PAV) [7] is an assisted ventilation mode which adjusts inspiratory pressure in proportion to the flow and volume generated by the patient. It was designed as an assisted ventilation mode to reduce the patient’s work of breathing and inspiratory effort, increasing the harmony between the patient and the machine. Proportional assist ventilation plus (PAV+) automatically adjusts the flow assist and volume assist to represent constant fractions of the measured resistance and elasticity of the patient’s respiratory system instantaneously. Theoretically, PAV can reduce the chance of respiratory muscle dysfunction and dys-synchrony events and increase the weaning success rate. Numerous recent studies have compared PAV to traditional PSV; however, data on this topic are still limited. A meta-analysis [8] supporting the clinical use of PAV was conducted; however, analysis of weaning outcomes for PAV is insufficient to make specific recommendations.

Therefore, we conducted a more comprehensive meta-analysis using state-of-the-art statistical methods. We applied trial sequential analysis (TSA) [9] to determine whether the included studies are conclusive and prevent premature conclusions from the meta-analysis. The aim is to compare PAV and PSV as weaning methods to facilitate liberation from mechanical ventilation and provide recommendations for clinical practice.

## Methods

We adhered to the Preferred Reporting Items for Systematic Reviews and Meta Analyses (PRISMA) [10] guidelines for performing systematic reviews and meta analyses of randomized controlled trials (RCTs) (Additional file 1: Table S1). This review protocol was prespecified in advance and registered with the Open Science Framework platform (protocol available at https://osf.io/jfe53).

### Data sources and searches

We searched PubMed, Embase, and the Cochrane Library to identify all relevant trials, by screening titles and reviewing abstracts, with no filters or language restrictions. In order to ensure that no RCTs were missing, conference abstracts and reference lists of included articles were reviewed. We searched for ongoing trials, using Google Scholar and the US government clinical trials database (www.ClinicalTrials.gov).

Two independent investigators (PHC and LJOY) conducted a systematic search for RCTs published up until April 2020, using the terms “Proportional Assisted Ventilation,” “Pressure Support Ventilation,” “Ventilation Weaning,” and “Spontaneous Breathing Trial” (Additional file 1: Table S2).

### Study selection

We selected RCTs that included adults (aged 18 years or older) with respiratory failure from various causes who had received invasive mechanical ventilation (MV) for 24 h or more and were undergoing weaning trials (using PAV, PAV+, or PSV) for liberation from ventilation. Studies published in English and Chinese language were selected.

The exclusion criteria were as follows: trials investigating neonatal or pediatric patients (patients aged less than 18 years), trials that extubated patients directly to noninvasive ventilation for weaning purposes, trials with no active comparison group (i.e., placebo or no treatment), and trials that compared the same ventilation mode but had different parameters.

### Data extraction and bias assessment

Two reviewers (HJJ and PHC) independently extracted the data from all included articles. Data extraction was performed to capture information on study-related, participant-related, and treatment-related characteristics. Authors of studies eligible for inclusion in our review were contacted if original data were missing.

The quality of the RCTs was appraised by HJJ and PHC using the Cochrane Risk of Bias tool [11]. Assessments of the risks of selection bias (random sequence generation, allocation concealment), performance bias (blinding of participants and personnel), detection bias (blinding of outcome assessment), attrition bias (incomplete outcome data), reporting bias (selective reporting), and other bias were appraised as high, unclear, and low risk (Additional file 1: Fig. S1). Any disagreement was resolved via group discussions [12].

### Outcome measures

We extracted data on one primary outcome and five secondary outcomes. The primary outcome was weaning success, defined as the absence of the requirement for invasive MV support, without reintubation, a cardiac arrest event, or mortality within 48 h after extubation (translaryngeal tube) or withdrawal (tracheostomy tube). The secondary outcomes were as follows: the proportion of patients requiring reintubation (defined as the patient requiring reintubation within 48 h after extubation), in-hospital mortality, ICU length of stay (the time from randomization to ICU discharge), weaning duration (the time from randomization to extubation), and ventilation duration (the time from intubation to extubation).

### Data synthesis and analysis

We analyzed the data as recommended in the Cochrane Handbook for Systematic Reviews of Interventions [13]. We analyzed dichotomous variables [14], using the Mantel-Haenszel method and DerSimonian-Laird estimator, and calculated risk ratios (RRs) with 95% confidence intervals (CIs). To measure continuous outcomes [15], we employed the inverse variance method and DerSimonian-Laird estimator, and calculated the mean difference (MD) with 95% CIs. Administration of fixed-effect or random-effects was interpreted with statistical heterogeneity by authors. If an estimate of the between-study variance, known as tau-squared, was low (or zero), then we would choose fixed-effect model; otherwise, we would choose random-effects. Heterogeneity was evaluated using the *I* square (*I*^2^) statistic [16] and Cochran’s Q test [17]. Statistically significant heterogeneity was defined as *I*^2^ > 50% and Cochran’s Q test *P* < 0.1. The presence of publication bias was assessed using funnel plots and Egger’s test [18].

We conducted subgroup analysis by the types of different proportional modes, PAV and PAV+, and examined the differences in outcomes between these subgroups by testing for interactions. We used a mixed-effects linear meta-regression model [19] to evaluate the cause of heterogeneity for all outcomes, with variables including mean age, sex, MV baseline duration, and physiology score. Sensitivity analyses were conducted by excluding the trials with a high risk of bias.

All statistical analyses were performed using the “metafor” [20] and “meta” packages of R software version 3.6.1 [21]. A *P* value of < 0.05 was taken to indicate statistical significance.

### Quality assessment

The Grading of Recommendations Assessment, Development, and Evaluation (GRADE) methodology [22] was used to assess the certainty of evidence from the included studies (GRADEpro, version 20; McMaster University, 2014).

### Trial sequential analysis

Sparse data and repetitive testing of accumulating data in meta-analyses can produce an increased risk of both type 1 and type 2 errors [23, 24]. Therefore, TSA was conducted to challenge the meta-analysis, in case the data were too sparse to confirm the conclusions, and avoid early overestimates by combining the estimated required information size with an adjusted threshold [25]. We constructed TSA boundaries, according to the O’Brien-Fleming alpha-spending function, to assess whether the *P* value was statistically significant enough to show the anticipated effect or whether the analysis should be terminated early [26].

A 5% (*α* = 0.05; two-sided) total risk of type 1 error and 80% statistical power were set. We assumed a relative risk reduction of 10% for the primary outcome of weaning success. The event rate in the control group was calculated from the mean of the event proportions in the low-risk-of-bias trials. We provide the TSA-adjusted 95% confidence intervals. Fixed-effect TSA was performed using trial sequential analysis software (version 0.9.5.10 Beta; Copenhagen Trial Unit, Copenhagen, Denmark).

## Results

**Fig. 1:**
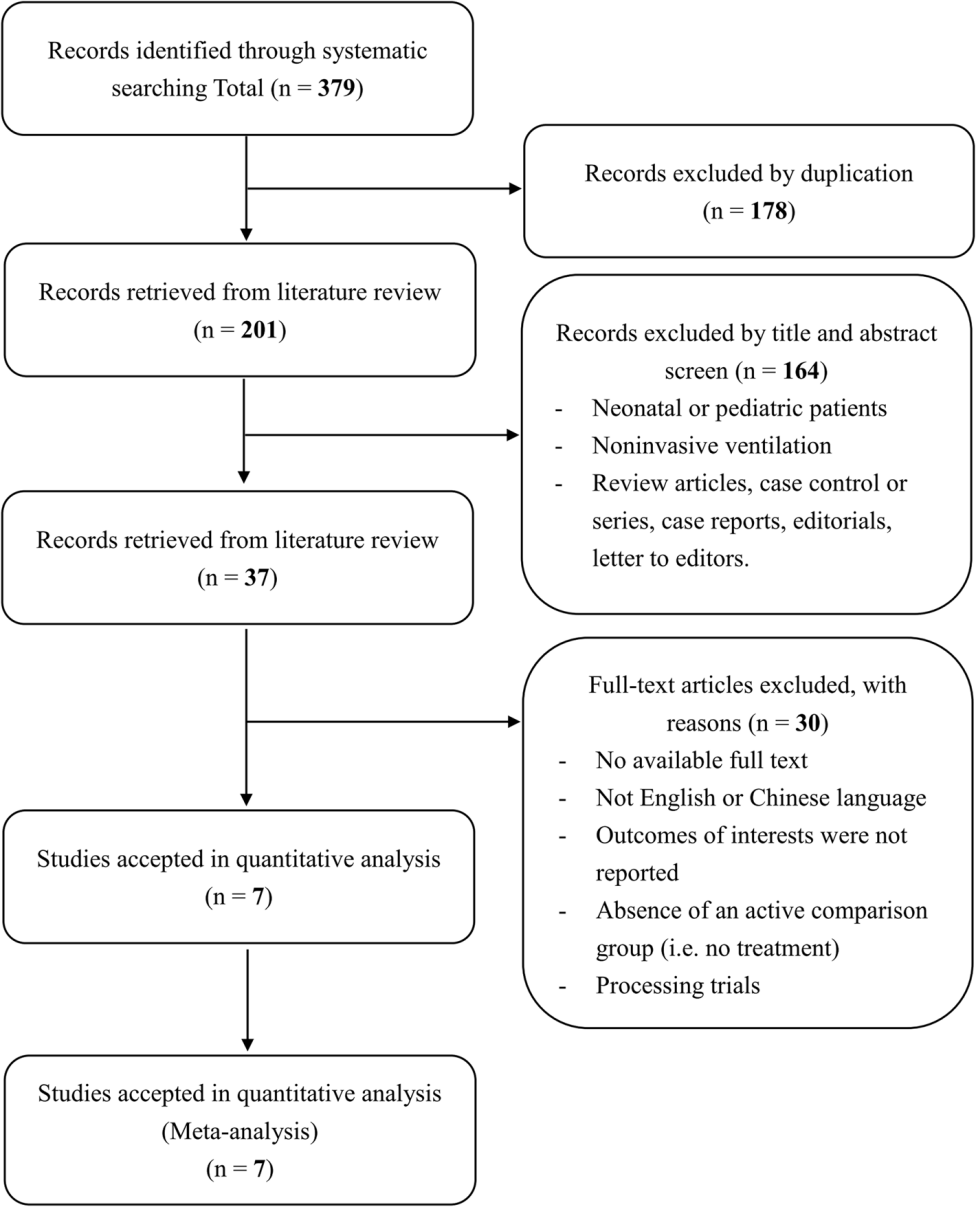
Flow diagram of the identification process for eligible studies

We screened 379 article titles and abstracts from the electronic databases, excluded 342 articles, and retrieved 37 articles for full-text assessment. The remaining seven studies [4, 27–32] (6 two-arm studies, 1 three-arm study) were included in our quantitative analysis. Figure 1 shows the flowchart of our literature search process to obtain eligible trials.

The included studies evaluated 634 patients randomized to two interventions: 326 patients receiving PAV as the weaning method and 308 receiving PSV. Summary individual-study characteristics and study-level patient characteristics from all included randomized trials are reported in Table 1 and Additional file 1: Table S3. The RCTs were published between 2008 and 2018. Five of these trials [4, 27, 28, 31, 32] included patients who were intubated due to a medical emergency, and two [29, 30] evaluated patients requiring mechanical ventilator support due to a medical or surgical cause. All patients received at least 24 h of invasive ventilator support. Three of the articles [27, 28, 32] did not report the mean MV duration. Two [28, 32] recorded no mean physiologic score.

**Table 1:** Characteristics of included studies

Authors	Year	Design	Country	Patient number	Age (mean)	Male (%)	Mean MV (days)	Mean PS	Intervention 1	Intervention 2
Xirouchaki et al. [4]	2008	RCT	Greece	208	60.9 (years)	66.3	4.0	APACHE II, 15.5	PAV+: initial assist 60–80%, reduced by 10–20% per hour. Extubation was performed at 10–20% assist.	PSV: initial PIP 20–25 cmH_2_O, reduced by 2–5 cmH_2_O per hour. Extubation was performed at PS 10–12 cmH_2_O.
Sasikumar et al. [27]	2013	RCT	India	23	48.6 (years)	69.6	NR	APACHE II, 20.7	PAV+: the protocol for PAV+ setting was not available.	PSV: the protocol for PSV setting was not available.
Elganady et al. [28]	2014	RCT	Egypt	60	59.7 (years)	81.7	NR	NR	PAV: initial assist 70%, reduced by 10–20% every 2 h. Extubation was performed at 10–20% assist.	PSV: initial PEEP 5 cmH_2_O, initial pressure support 5~8 cmH_2_O. No reduction during the trial. Extubation was performed at 120 min.
Teixeira et al. [29]*	2015	RCT	Brazil	160	44.5 (years)	65.6	6.6	APACHE II, 22.7	PAV+: initial assist and the method for reduction were not available. Extubation was performed at 40% assist.	PSV: pressure support with 7 cmH_2_O. No reduction method was mentioned. The timing for extubation was not available.
Bosma et al. [30]	2016	RCT	Canada	50	64.8 (years)	50.0	5.8	APACHE II, 26.5	PAV+: initial assist 70%, reduced by the judgment of a respiratory therapist every 2 to 3 h.	PSV: initial PS 15 cmH_2_O, reduced by the judgment of a respiratory therapist every 2 to 3 h.
Botha et al. [31]	2018	RCT	Australia	50	63.2 (years)	59.2	3.4	APACHE III, 76.7; SASP, 45.5	PAV+: initial assist 70%, reduced by 10%. Extubation was performed at 30% assist.	PSV: initial PS was set at patient’s need; the reduction method was not mentioned. Extubation was performed at PS 10 cmH_2_O.
Salama et al. [32]	2018	RCT	Egypt	150	NA	NA	NA	NA	PAV+	PSV

*Three-arm study

### Primary outcome: weaning success

**Fig. 2:**
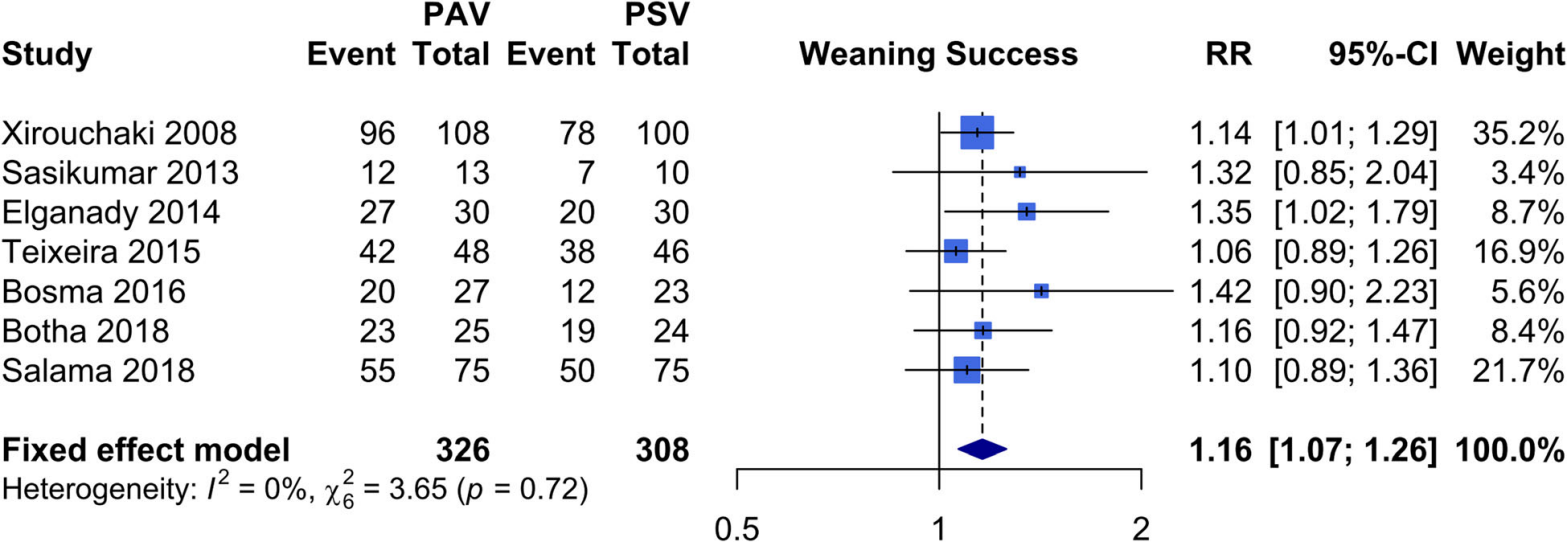
Meta-analysis of weaning success. PAV, proportional assisted ventilation; PSV, pressure support ventilation; RR, risk ratio; CI, confidence interval

Seven studies [4, 27–32] with 634 patients were included in the analysis. As the tau-squared was zero in current meta-analysis, we applied fixed-effect model. The rate of weaning success was significantly greater in patients undergoing PAV compared to patients undergoing PSV (fixed-effect, RR 1.16; 95% CI 1.07–1.26; *I*^2^ = 0.0%, Cochran’s Q *P* value 0.72) (Fig. 2). The quality assessment using the GRADE approach was moderate (Additional file 1: Table S4).

### Secondary outcomes (Table 2)

Compared with PSV, PAV was associated with a lower proportion of patients requiring reintubation (6 studies [4, 27–31], *n* = 484 patients, fixed-effect, RR 0.49; 95% CI 0.28–0.87; *I*^2^ = 0%, Cochran’s Q *P* value 0.81), a shorter ICU length of stay (5 studies [27–31], *n* = 276 patients, fixed-effect, MD − 1.58 (days); 95% CI − 2.68 to − 0.47; *I*^2^ = 0%, Cochran’s Q *P* value 0.76), and a shorter total duration of ventilation (3 studies [27, 28, 30], *n* = 133 patients, fixed-effect, MD − 40.26 (hours); 95% CI − 66.67 to − 13.84; *I*^2^ = 0%, Cochran’s Q *P* value 0.56). There was no significant difference between PAV and PSV in terms of mortality (5 studies [4, 28–31], *n* = 461 patients, fixed-effect, RR 0.66; 95% CI 0.42–1.06; *I*^2^ = 0%, Cochran’s Q *P* value 0.56) or weaning duration (3 studies [27, 30, 31], *n* = 123 patients, fixed-effect, MD − 0.01 (hours); 95% CI − 1.30–1.28; *I*^2^ = 0%, Cochran’s Q *P* value 0.57).

**Table 2:** Meta-analysis of secondary outcomes

Comparison	Trials (***n***)	Secondary outcomes	Summary estimate with 95% CI	***I***^**2**^ (%)	Cochran’s Q ***P*** value
PAV versus PSV for weaning from mechanical ventilation in adults	6 (484)	Reintubation	RR, 0.49 [0.28; 0.87]*	0	0.81
	5 (461)	Mortality	RR, 0.66 [0.42; 1.06]	0	0.56
	5 (276)	ICU length of stay (days)	MD, − 1.58 [− 2.68; − 0.47]*	0	0.76
	3 (122)	Duration of weaning (hours)	MD, − 0.01 [− 1.30; 1.28]	0	0.57
	3 (133)	Duration of ventilation (hours)	MD, − 40.26 [− 66.67; − 13.84]*	0	0.56

1. Values of RR less than 1 indicates a reduction in risk for the events with the PAV group

2. Summary estimate presents the result of fixed-effect meta-analysis

*Statistically significant

### Meta-regression, subgroup analyses, sensitivity analyses, and publication bias

The meta-regression analysis examined the relationship between the following four variables: mean age, sex, baseline duration of MV, physiology score, and all of the outcomes (Additional file 1: Table S5). The meta-regression analysis showed no difference in interactions of all outcomes with overall variables.

A subgroup analysis was conducted of the different types of PAV. In the subgroup of patients undergoing PAV+, we found a greater chance of weaning success with low heterogeneity (fixed-effect, RR 1.14; 95% CI 1.05–1.24; *I*^2^ = 0%). A similar trend was seen in the subgroup of patients undergoing PAV (fixed-effect, RR 1.35; 95% CI 1.02–1.79; *I*^2^ = not applicable as there was only one included study) (Additional file 1: Fig. S2).

We also performed a sensitivity analysis, removing high risk of bias studies, and the results did not change substantially (Additional file 1: Fig. S3). There was no publication bias by applying the funnel plots and Egger’s test (Additional file 1: Fig. S4). However, it should be interpreted with caution, because too few studies were included in our meta-analysis for assessing publication bias [33, 34].

### Trial sequential analysis

**Fig. 3:**
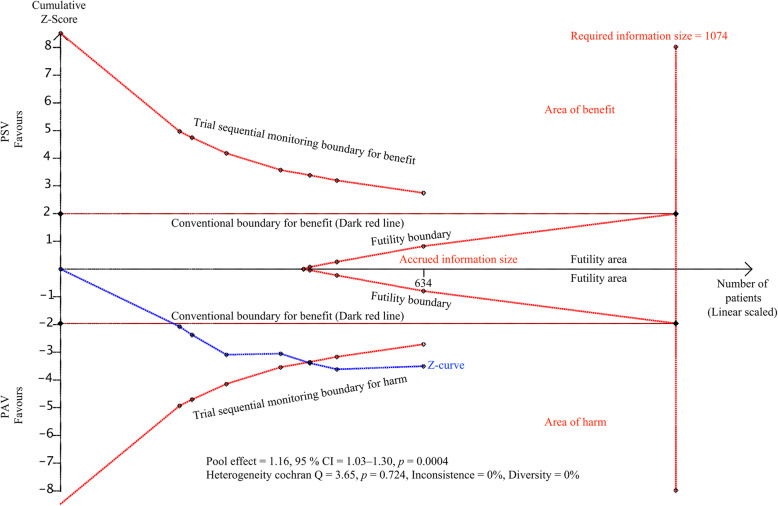
Trial sequential analysis of weaning success. The *x*-axis represents the accrued versus required information size of patients. The *y*-axis represents the *z* values, representing the accumulating statistical information. The blue line (*z*-curve) represents the cumulative *Z* value, and each square represents an individual trial. The small red lines at the top and bottom left-hand corners, trial sequential boundaries for benefit or harm, represent the threshold for statistical significance in TSA. The horizontal dark red lines represent the threshold for significance in conventional meta-analysis, at 1.96 of the *Z* value, corresponding to 0.05 of the *P* value. The red line in a triangle shape represents the futility boundaries and futility area in TSA. In this figure, TSA of the primary outcome, weaning success, showed that the *Z*-curve has not yet reached the required information size, but crosses the conventional boundary and the trial sequential monitoring boundary curve for benefit with statistical significance, suggesting conclusive and robust results in favor of PAV

Figure 3 shows the TSA of the primary outcome, weaning success. In this TSA, the required information size was calculated allowing for an overall type 1 error of 5%, a type 2 error of 20%, and assuming a control event proportion of 0.72, a relative risk reduction of 10% for the effect size, and a model variance based estimate of diversity. The estimates for the control event proportion and diversity were taken from the included trials, as described in the “Methods” section.

TSA of weaning success showed that the effect size of 634 did not exceed the required information size of 1074 patients. The TSA-adjusted confidence interval was RR 1.16 (95% CI 1.03, 1.30). When the accumulated data were less than the required information size, the cumulative *z*-curve needed to cross the monitoring boundaries to declare either significance or futility. The cumulative *z*-curve crossed the conventional boundary and the trial sequential monitoring boundary for benefit in favor of PAV, suggesting conclusive, robust results. Therefore, TSA confirmed our meta-analysis providing convincing statistical evidence. The TSA results of all secondary outcomes demonstrated inconclusive results with sparse data, which are described in Additional file 1: Fig. S5.

## Discussion

This meta-analysis compared the efficacy of PAV and PSV as weaning methods in mechanically ventilated patients. Patients undergoing PAV demonstrated a higher chance of weaning success compared to patients undergoing PSV. These results were shown to be sufficient and conclusive using TSA. Our results also revealed a lower reintubation rate and a shorter ICU length of stay in patients undergoing PAV compared to patients undergoing PSV.

Patient-ventilator dys-synchrony is a common phenomenon in mechanically ventilated patients. Dys-synchronies, such as insufficiency, work of breathing, and double triggering, have negative effects on patients’ respiratory systems, which lead to delayed weaning off MV. The patient’s timing of inspiration and expiration does not always meet the ventilator trigger, causing respiratory muscle fatigue and hampering the patient from successful weaning [35]. PAV was proposed as a powerful means of improving patient-ventilator interaction and was designed to adjust inspiratory pressure proportionally to the patient’s inspiratory demand. PAV generates pressure in proportion to the patients’ instantaneous breathing effort, providing timely adjustment, thereby improving the patient-ventilator relationship [6]. Through reducing patients’ work of breathing and saving their physical energy, they can wean more efficiently, thereby improving the weaning success rate and reducing the length of ICU and hospital stay [8]. This study investigated the efficacy of PAV as a weaning mode, compared to PSV. We found that ventilated patients undergoing PAV had a higher weaning success rate and a shorter ICU stay. Nevertheless, the lack of simple and reliable software to estimate the patient-ventilator relationship in PAV in complex and dynamic clinical environments remains an obstacle to overcome.

PAV may offer several physiological benefits, including reduction of ineffective efforts, and improved quality of sleep [36], arterial blood gas tension [6], and patient-ventilator interaction [37]. Previous studies demonstrated that PAV could reduce the sedative medication dose because of better patient-ventilator interaction [30]. Using a lower dose of sedative medication is associated with shorter MV duration. In one meta-analysis performed by Kataoka et al. [38], the use of proportional mode was significantly associated with reduced patient-ventilator dys-synchrony, the rate of weaning failure, and the duration of mechanical ventilation compared with PSV. However, Kataoka et al. did not compare the mortality rate between proportional mode and PSV. While Kataoka et al. [38] compared proportional mode with PSV, we focused on the comparison between PAV and PSV. Three more studies were included in our meta-analysis. We also performed trial sequential analysis to reduce both type 1 and type 2 errors, avoiding overestimates, and consolidating our result. In another meta-analysis [8], the evidence supporting clinical use of PAV as a weaning modality was assessed. The authors found that PAV resulted in an insignificant reduction in weaning time and had no effect on mortality reduction or reintubation rate compared to PSV. In our analysis, PAV did not reduce the weaning time or the mortality rate but was associated with a significantly decreased reintubation rate, which was inconsistent with the previous study’s results [8].

PAV had a lower but insignificantly reduced risk of mortality (RR 0.66; 95% CI 0.42–1.06; *I*^2^ = 0%) compared to PSV. In this study, the mortality rates in patients undergoing PAV and PSV were 10.0% and 15.1%, respectively. Therefore, this finding may due to a limited number of cases (*n* = 461) with relatively low mortality. Furthermore, mechanical ventilation is provided not only for respiratory reasons but also for unstable and comatose patients. Organ failure represents the main cause of death in a general ICU population. PAV can reduce the duration of ventilation but may not change the severity of the underlying diseases in patients on mechanical ventilation. Future large-scale studies or analyses are needed to confirm this result. A recent expert consensus guideline [39] highlights the growing recognition that initial SBT with pressure augmentation may increase extubation success and reduce ICU mortality. TSA partially confirmed this benefit, and the findings of our analysis may provide a direction for future studies to investigate the beneficial effects of PAV.

The results of this meta-analysis might be elucidated in light of its strengths and limitations. When the data were insufficient to reduce the risk of misinterpreting random error [40], TSA provided more information around imprecision and added a new dimension to yield firm conclusions. With this strict approach, the benefit of using PAV to improve weaning success remained statistically significant, even though the required information size had not yet been met. Furthermore, the GRADE methodology was conducted to qualify the evidence of the results through critical appraisal of the included studies.

Clinical heterogeneity exists in our included studies. Critically ill patients with cardiogenic or respiratory dysfunction, neurological diseases, and traumatic settings were enrolled in this study. These confounding factors should be considered cautiously when interpreting our results. Another limitation was that not every outcome of interest was recorded in each of the included RCTs, and insufficient data hinder comprehensive subgroup analysis. Furthermore, PAV and PSV are different respiratory modes. Blinding participants and personnel may not have been possible, increasing the risk of bias. Lastly, TSA of secondary outcomes showed inconclusive results, meaning that the risk of spurious findings remains. More high-quality randomized control should be conducted to provide reasonably firm evidence of a conferred benefit for patients.

## Conclusions

In conclusion, our meta-analysis of the current evidence suggests using PAV, as a weaning method, improves the weaning success rate, compared to PSV. The evaluation of the reintubation rate, mortality rate, length of stay in ICU, duration of MV, and weaning duration generally favors using PAV. Further investigation is needed to better understand the effect of proportional ventilation on these outcomes.

## Supplementary information

**Additional file 1: Table S1.** PRISMA Checklist. **Table S2.** Search strategy in MEDLINE (Ovid), Embase, and Cochrane Library. **Table S3.** Detailed information of the included trials. **Table S4.** GARDE, Summary of findings. **Table S5.** Meta-regression analysis of outcomes with variables (Mean Age, Gender, Baseline duration of mechanical ventilation, and Physiology score). **Fig. S1.** Assessment of risk of bias with the Cochrane Risk of Bias tool. **Fig. S2.** Subgroup analysis of outcomes by the types of different proportional modes. **Fig. S3.** Sensitivity analyses of outcomes were conducted by excluding the trials with a high risk of bias. **Fig. S4.** Funnel plots and Egger’s test for detecting the publication bias. **Fig. S5.** Trial sequential analysis of secondary outcomes.

## Data Availability

The datasets used during the current study are available from the corresponding author on reasonable request.

## Abbreviations

APACHE II: Acute Physiology and Chronic Health Evaluation II score
CI: Confidence interval
COPD: Chronic obstructive pulmonary disease
GRADE: The Grading of Recommendations Assessment, Development, and Evaluation
ICU: Intensive care unit
MD: Mean difference
MV: Mechanical ventilation
NA: Not available
PAV: Proportional assist ventilation
PAV+: Proportional assist ventilation plus
PEEP: Positive end-expiratory pressure
PIP: Peak inspiratory pressure
PRISMA: Preferred Reporting Items for Systematic reviews and Meta-analysis
PSV: Pressure support ventilation
RR: Risk ratio
RCTs: Randomized controlled trials
SBT: Spontaneous breathing trial
TSA: Trial sequential analysis
