# Supplementary material for: Proportional assist ventilation versus pressure support ventilation for weaning from mechanical ventilation in adults: a meta-analysis and trial sequential analysis
Source: Crit Care. 2020 Sep 14;24:556. doi: 10.1186/s13054-020-03251-4 (PMC7487443; doi:10.1186/s13054-020-03251-4)
Supplement: Supplementary file 1 — Additional file 1: Table S1. PRISMA Checklist. Table S2. Search strategy in MEDLINE (Ovid), Embase, and Cochrane Library. Table S3. Detailed information of the included trials. Table S4. GARDE, Summary of findings. Table S5. Meta-regression analysis of outcomes with variables (Mean Age, Gender, Baseline duration of mechanical ventilation, and Physiology score). Fig. S1. Assessment of risk of bias with the Cochrane Risk of Bias tool. Fig. S2. Subgroup analysis of outcomes by the types of different proportional modes. Fig. S3. Sensitivity analyses of outcomes were conducted by excluding the trials with a high risk of bias. Fig. S4. Funnel plots and Egger’s test for detecting the publication bias. Fig. S5. Trial sequential analysis of secondary outcomes. [file 13054_2020_3251_MOESM1_ESM.pdf]

Supplementary Information - Additional file 1

Proportional assist ventilation versus pressure support ventilation for weaning from mechanical ventilation in adults: A Meta-analysis and Trial sequential analysis

Liang-Jun Ou-Yang MD, Po-Huang Chen MD, Hong-Jie Jhou MD, Vincent Yi-Fong Su MD, Cho-Hao Lee MD

Contents

Table S1. PRISMA Checklist

Table S2. Search strategy

Table S3. Detailed information of the included trials

Table S4. GARDE, Summary of findings

Table S5. Meta-regression analysis

Figure S1. Assessment of risk of bias

Figure S2. Subgroup analysis of outcomes

Figure S3. Sensitivity analysis of outcomes

Figure S4. Funnel plots and Egger’s test

Figure S5. Trial sequential analysis of secondary outcomes

Table S1. PRISMA Checklist

Section/topic	Item No	Checklist item	Reported on page No
**Title**			
Title	1	Identify the report as a systematic review, meta-analysis, or both	1
**Abstract**			
Structured summary	2	Provide a structured summary including, as applicable, background, objectives, data sources, study eligibility criteria, participants, interventions, study appraisal and synthesis methods, results, limitations, conclusions and implications of key findings, systematic review registration number	3
**Introduction**			
Rationale	3	Describe the rationale for the review in the context of what is already known	4
Objectives	4	Provide an explicit statement of questions being addressed with reference to participants, interventions, comparisons, outcomes, and study design (PICOS)	5
**Methods**			
Protocol and registration	5	Indicate if a review protocol exists, if and where it can be accessed (such as web address), and, if available, provide registration information including registration number	5
Eligibility criteria	6	Specify study characteristics (such as PICOS, length of follow-up) and report characteristics (such as years considered, language, publication status) used as criteria for eligibility, giving rationale	6
Information sources	7	Describe all information sources (such as databases with dates of coverage, contact with study authors to identify additional studies) in the search and date last searched	5-6
Search	8	Present full electronic search strategy for at least one database, including any limits used, such that it could be repeated	6
Study selection	9	State the process for selecting studies (that is, screening, eligibility, included in systematic review, and, if applicable, included in the meta-analysis)	6
Data collection process	10	Describe method of data extraction from reports (such as piloted forms, independently, in duplicate) and any processes for obtaining and confirming data from investigators	6
Data items	11	List and define all variables for which data were sought (such as PICOS, funding sources) and any assumptions and simplifications made	7

Section/topic	Item No	Checklist item	Reported on page No
Risk of bias in individual studies	12	Describe methods used for assessing risk of bias of individual studies (including specification of whether this was done at the study or outcome level), and how this information is to be used in any data synthesis	6-7
Summary measures	13	State the principal summary measures (such as risk ratio, difference in means).	3, 7
Synthesis of results	14	Describe the methods of handling data and combining results of studies, if done, including measures of consistency (such as I^2 statistic) for each meta-analysis	8
Risk of bias across studies	15	Specify any assessment of risk of bias that may affect the cumulative evidence (such as publication bias, selective reporting within studies)	8
Additional analyses	16	Describe methods of additional analyses (such as sensitivity or subgroup analyses, meta-regression), if done, indicating which were pre-specified	8-9
**Results**			
Study selection	17	Give numbers of studies screened, assessed for eligibility, and included in the review, with reasons for exclusions at each stage, ideally with a flow diagram	9
Study characteristics	18	For each study, present characteristics for which data were extracted (such as study size, PICOS, follow-up period) and provide the citations	10
Risk of bias within studies	19	Present data on risk of bias of each study and, if available, any outcome-level assessment (see item 12).	S6
Results of individual studies	20	For all outcomes considered (benefits or harms), present for each study (a) simple summary data for each intervention group and (b) effect estimates and confidence intervals, ideally with a forest plot	S5
Synthesis of results	21	Present results of each meta-analysis done, including confidence intervals and measures of consistency	11
Risk of bias across studies	22	Present results of any assessment of risk of bias across studies (see item 15)	S5
Additional analysis	23	Give results of additional analyses, if done (such as sensitivity or subgroup analyses, meta-regression) (see item 16)	13-14, S3-4, S8

Section/topic	Item No	Checklist item	Reported on page No
**Discussion**			
Summary of evidence	24	Summarise the main findings including the strength of evidence for each main outcome; consider their relevance to key groups (such as health care providers, users, and policy makers)	14-15
Limitations	25	Discuss limitations at study and outcome level (such as risk of bias), and at review level (such as incomplete retrieval of identified research, reporting bias)	16
Conclusions	26	Provide a general interpretation of the results in the context of other evidence, and implications for future research	16-17
**Funding**			
Funding	27	Describe sources of funding for the systematic review and other support (such as supply of data) and role of funders for the systematic review	18

Table S2. Search strategy

MEDLINE (Ovid) search strategy to April, 2020

(((((("weaning"[All Fields] OR "spontaneous breathing test"[All Fields]) OR "spontaneous breathing trial"[All Fields]) OR "sbt"[All Fields]) OR "spontaneous breathing"[All Fields]) OR (("ventilator"[All Fields] OR "mechanical ventilation"[All Fields]) OR "ventilation"[All Fields])) AND (("pressure support"[All Fields] OR "pressure support mode"[All Fields]) OR "pressure support ventilation"[All Fields])) AND ((((("proportional assist ventilation"[All Fields] OR "proportional assist ventilator"[All Fields]) OR "proportional assist"[All Fields]) OR "proportional assisted ventilation"[All Fields]) OR "PAV"[All Fields]) OR "PAV+"[All Fields])

Embase search strategy to April, 2020

("weaning"/exp OR "weaning" OR "spontaneous breathing test" OR "spontaneous breathing trial"/exp OR "spontaneous breathing trial" OR "sbt" OR "spontaneous breathing"/exp OR "spontaneous breathing" OR "ventilator"/exp OR "ventilator" OR "mechanical ventilation"/exp OR "mechanical ventilation" OR "ventilation"/exp OR "ventilation") AND ("pressure support" OR "pressure support mode" OR "pressure support ventilation"/exp OR "pressure support ventilation") AND ("proportional assist ventilation"/exp OR "proportional assist ventilation" OR "proportional assist ventilator" OR "proportional assist" OR "proportional assisted ventilation" OR "pav" OR "pav+")

Cochrane Library search strategy to April, 2020

(("weaning"[All Fields] OR "spontaneous breathing test"[All Fields] OR "spontaneous breathing trial"[All Fields] OR "sbt"[All Fields] OR "spontaneous breathing"[All Fields]) OR ("ventilator"[All Fields] OR "mechanical ventilation"[All Fields] OR "ventilation"[All Fields]) AND ("pressure support"[All Fields] OR "pressure support mode"[All Fields] OR "pressure support ventilation"[All Fields]) AND ("proportional assist ventilation"[All Fields] OR "proportional assist ventilator"[All Fields] OR "proportional assist"[All Fields] OR "proportional assisted ventilation"[All Fields] OR "PAV"[All Fields] OR "PAV+"[All Fields]))

Table S3. Detailed information of the included trials

Xirouchaki 2008

Methods: Single center randomized controlled trial

Patients: 208 adult patients in medical-surgical ICU, under invasive mechanical ventilation for at least 36 hours and ventilated with a pressure or a volume controlled mode.

- Mean age: 60.9 years old, male: 66.34%
- Mean duration of mechanical ventilation: 4 days
- Mean severity: APACHE II: 15.48
- Reason for intubation: ARDS or sepsis (30.8%), trauma with brain injury (11.53%), trauma without brain injury (12.81%)
- Comorbidities: CNS diseases excluding trauma (11.5%), cardiogenic shock or CHF (4.8%), AECOPD (4.8%)

Interventions: Puritan-Bennett 840 ventilator

- Intervention: PAV+, the assist was started at 60-80% and was reduced by 10-20% every 1 hour with monitoring of respiratory distress. Extubation was performed once no respiratory distress occurred at 10-20% assist, $PEEP_E \leq 5$ cmH_2O, and $FiO_2 \leq 50\%$.
- Comparison: PSV, the inspiratory pressure was set to 20-25 cmH_2O (including $PEEP_E$) and was reduced by 2-5 cmH_2O every 1 hour with monitoring of respiratory distress. Extubation was performed once no respiratory distress occurred at PS ≤10-12 cmH_2O, $PEEP_E \leq 5$ cmH_2O, and $FiO_2 \leq 50\%$.

Outcomes:

- Weaning failure: defined as reintubation required within 48 hours of extubation
- The incidence of patient-ventilator dyssynchronies

Notes

Study protocol

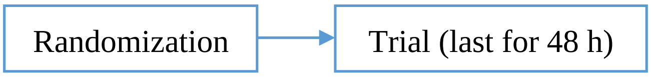

- The trial would be halted if the patient needed to receive a procedure that required total sedation, and PAV+ or PSV was re-instituted once the inclusion was met again.
- Sedation and analgesia were allowed during the trial.
  - Remifentanyl was used for analgesia and propofol for sedation.
  - Vasopressors (mainly norepinephrine) were given following usual clinical guidelines.

Inclusion criteria:

- Be able to trigger the ventilator at a satisfactory rate (>10 breaths/min)
- Adequate oxygenation: PaO_2 >60 mmHg, with FiO_2 < 65%
- $PEEP_{TOT}$ <15 cmH_2O
- No severe acidemia (pH >7.30)
- No severe hemodynamic instability
- No severe bronchospasm: end-inspiratory airway resistance (Rmin) measured during CMV < 20 cmH_2O/l/sec

	• Stable neurological status: no need for heavy sedation to control intracranial pressure or for any intervention during the previous 24 hours either to lower intracranial pressure to normal values (≤12 cmH_2O) or to manage any event related to CNS (i.e. seizures).
Exclusion criteria	• A do-not-resuscitate order<br>• Mechanical ventilation with assisted modes (independent on the duration)<br>• Expected poor short-term prognosis (< 3 months)<br>• Neuromuscular disease with respiratory muscle involvement that could permanently impair the ability to breathe spontaneously<br>• Age < 18 and > 85 years.
Failure criteria (Patients who met these criteria would be withdrawn from the study.)	• Respiratory distress despite adjustment of $PEEP_E$ and/or assist level<br>• Hypoxemia (SaO_2 <90%) despite adjustment of FiO_2 and/or $PEEP_E$ and/or assist level<br>• Hypercapnia with acidemia (pH <7.35 or pH <7.30 in patients with pre-existing metabolic acidosis) despite adjustment of sedation level and/or $PEEP_E$ and/or assist level<br>• Severe hemodynamic instability (need for norepinephrine >0.5 μg/kg/h) or arrhythmias<br>• Acute ischemic heart disease<br>• Increased need for sedation for medical reasons (i.e. CNS disease, agitation, fighting the ventilator) that results in depressed respiratory drive<br>• The need for reintubation in less than 48 h after extubation in patients in whom extubation was performed within the 48-h study period (extubation failure)

Note:

APACHE II, Acute Physiology and Chronic Health Evaluation score II; ARDS, acute respiratory distress syndrome; CHF, congestive heart failure; CNS, central nervous system; AECOPD, acute exacerbation of chronic obstructive pulmonary disease.

FiO_2, fractional concentration of inspired O_2; $PEEP_{TOT}$, total positive end-expiratory pressure, including $PEEP_E$, extrinsic positive end-expiratory pressure, and $PEEP_I$, intrinsic positive end-expiratory pressure;

Respiratory distress was defined as respiratory rate > 35b/min, tidal volume ≥5 ml/kg at SaO2 ≥90%.

Sasikumar 2013

Methods

Single center randomized controlled trial

Patients

23 adult patients in medical-surgical ICU, under invasive mechanical ventilation for at least 48 hours and ventilated with assist/control (A/C) or synchronized intermittent mandatory ventilation (SIMV)

- Mean age: 48.57 years old; Male: 69.57%
- Mean duration of mechanical ventilation: not available
- Mean severity: APACHE II: 20.74
- Reason for intubation: ARDS (43.48%), Sepsis (34.78%)

Interventions

Puritan-Bennett 840 ventilators

- Intervention: PAV+, the protocol for PAV+ setting was not available.
- Comparison: PSV, the protocol for PSV setting was not available.

Outcomes

- Length of ICU stay
- Duration of weaning
- Day to extubate

Notes

Study protocol

30-min PSV as SBT → Randomization → 30-min washout → Trial

- All patients were assessed for readiness for weaning using SBT criteria as listed in the “inclusion criteria” cell.
- 2 sets of ABG were obtained: first set was to confirm the readiness for SBT.
- A 30-minute PSV trial was given to all patients before further grouping as SBT.
- After randomization, a wash-out time of 30 minutes was given for patients in either group to nullify the effect of previous PSV mode.

Inclusion criteria

- Age >18 years
- No motor neuron disease, neuromuscular disease, COPD, or end stage diseases
- Spontaneous breathing efforts
- Adequate oxygenation: $PaO_2/FiO_2 \geq 150$, $FiO_2 < 50\%$, PEEP <8 cmH_2O
- No hypothermic (≤36.6℃) nor hyperthermic (>38℃)
- No excessive tracheal secretions (require suctioning <4 times a day)
- No pulmonary hemorrhage
- No coagulopathy (platelet count <50,000 cells/mm^3)
- Stable neurological status: no sedative drugs used
- Hemodynamic stability: no clinically important hypotension and no requirement for vasopressors or a requirement only for low-dose vasopressor therapy (e.g. dopamine ≤5 μg/kg/min)

Exclusion criteria

- Patients <18 years
- Body weight <25 kg
- Patients on ventilator only for airway protection

	• Progressive motor neuron disease<br>• Neuromuscular disease (including anticholinesterase poisoning)<br>• Chronic obstructive pulmonary disease (emphysema, dynamic hyperinflation, presence of bullae)<br>• Patients diagnosed with end stage disease
Failure criteria (Patients who met these criteria would be withdrawn from the study.)	• Spontaneous respiratory rate of ≥35 b/min or <10 b/min<br>• Tidal volume below <6-8 ml/kg<br>• Any increase or decrease in blood pressure by 20 mm Hg systolic pressure or a systolic pressure >180 mm Hg<br>• Deterioration in sensorium (restless or drowsy)<br>• SaO2 ≤ 90%, PaO2 <80 mm Hg on FiO2 ≤0.5<br>• Hypercapnia (PaCO2 >45 mm Hg or >20% from pre-extubation)<br>• Acidemia (pH <7.33)<br>• Clinical signs of respiratory muscle fatigue or increased work of breathing, such as excessive diaphoresis, intercostal retractions, tracheal tug, paradoxical breathing pattern, use of accessory muscles and nasal flaring

Note:

b/min: breaths per minute (respiratory rate) or beats per minute (heart rate); SBT: spontaneous breathing trial; COPD, chronic obstructive pulmonary disease;

Elganady 2014

Methods: Single center randomized controlled trial

Patients: 60 adult patients diagnosed with acute exacerbation of COPD in medical ICU, under invasive mechanical ventilation for at least 24 hours. The ventilator mode used was not available.

- Mean age: 59.67 years old; male: 81.67%
- Reason for intubation: all patients had COPD, the precipitating factors of intubation included chest infection (95%), and irritants exposure (5%).

Interventions: Galileo GOLD ventilators

- Intervention: PAV, the assist was started at 70% and was reduced by 10-20% every 2 hours with monitoring of respiratory distress. Extubation was performed once no respiratory distress occurred at 10-20% assist.
- Comparison: PSV, start SBT with low level of PEEP (e.g., 5 cmH_2O) and low level of pressure support (e.g., 5-8 cmH_2O). There was no support reduction. Extubation was performed if no sign of respiratory distress at 120 minutes.

Outcomes:

- Weaning success: absence of tachypnea >35 b/min, tachycardia >120 b/min, PaO_2/FiO_2 >150, hemodynamic stability (no need for vasopressors or a requirement only for low-dose vasopressors, such as dopamine ≤5 μg/kg/min)
  - Extubation failure was defined as respiratory distress, hemodynamic instability, reintubation within 72 hours after extubation.
- Duration of mechanical ventilation
- Length of stay in ICU and in hospital
- 28-day mortality rate

Notes

Study protocol

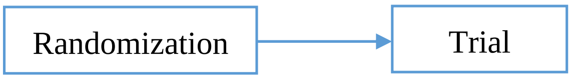

- Patients were randomized into group A (PAV mode) and group B (PSV mode).
- Patients in the PSV group could fully rest until the next day when the process began again if they were unable to tolerate or distressed.

Inclusion criteria:

- Reversal of the cause of mechanical ventilation.
- Hemodynamic stability: no clinically important hypotension and no requirement for vasopressors or a requirement only for low-dose vasopressor therapy (e.g. dopamine ≤5 μg/kg/min)
- Patient is capable of initiating an inspiratory effort.
- Adequate oxygenation: PaO_2 >60 mmHg with FiO_2 <40% PaO_2/FiO_2 >150–200 mmHg, required positive end expiratory pressure (PEEP) <5-8 cm H_2O
- Heart rate <120, respiratory rate <35, pH >7.35, tidal volume (VT) >5 ml/kg, rapid shallow breathing index (RSBI) (respiratory rate/tidal volume) <105, minute volume < 10 L/min.
- No electrolyte disturbances, no sedation or narcotics.

	• Good nutritional status and no clinically evident myopathy or neuropathy.<br>• Corrected reversible causes of weaning failure such as sepsis or heart failure.
Exclusion criteria	• Pregnancy<br>• Hemodynamic instability<br>• Severe neurological disease hindering the respiratory drive<br>• Patients on mechanical ventilation for less than 24 h (including self extubation or death).
Failure criteria	Not available.

Note:

Respiratory distress was defined as heart rate >120% of the usual rate for >5 min and/or systolic blood pressure >180 or <90 mmHg and/or systolic BP changes >20% of the previous value for >5 min, RR >40 bpm for >5 min, marked use of respiratory muscles, diaphoresis, abdominal paradox, or patient complaints of dyspnea.

Teixeira 2015

Methods: Single center randomized controlled trial

Patients: 160 adult patients in medical ICU, under invasive mechanical ventilation for at least 24 hours. The ventilator mode used was not available.

- Mean age: 44.5 years old; male: 65.60%
- Mean duration of mechanical ventilation: 6.6 days
- Mean severity: APACHE II: 22.7
- Reason for intubation: traumatic brain injury (27.5%), trauma without brain injury (17.5%), neurological diseases (12.5%), post-operation (23.13%)
- Comorbidities: COPD (22.5%), CHF (16.88%), obesity (19.38%).

Interventions: Puritan-Bennett 840 ventilators or Inter 7 Plus ventilator (while using PSV)

- Intervention: PAV+, initial assist was not available. Success was defined as subjects remaining in the comfort zone with assist ≤40% support.
- Comparison 1: T-piece, with supplemental oxygen to maintain SpO_2 >92%
- Comparison 2: PSV, Pressure support with 7 cmH_2O

Outcomes:

- Weaning success: extubation failure was defined as reintubation within 48 hours.
- Duration of mechanical ventilation
- Length of stay in ICU and in hosiptal

Notes

Study protocol

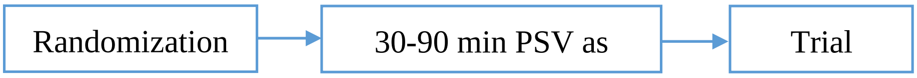

- A 30-90 minutes PSV trial was given to all patients as SBT.
- If the patients had intolerance signs, they would rest and be re-evaluated in 24 hours.

Inclusion criteria:

- Improvement or resolution of the cause that led to acute respiratory failure
- PaO_2 ≥60 mmHg with FiO_2 ≤45%,
- PaO_2/FiO_2 >200 mmHg
- PEEP ≤8 cmH2O
- Glasgow coma scale score ≥9
- Peripheral temperature <38℃
- Low doses of vasoactive drugs
- Hemodynamic stability

Exclusion criteria:

- Tracheostomy
- Death without weaning
- Self-extubation
- Extubation by clinical decision (decided and performed by staff, not meeting protocol requirements)
- Presence of progressive neuromuscular disease
- Spontaneous ventilation maintenance

Failure criteria*:

- Respiratory rate >35 b/min

- SpO_2 <90%
- Heart rate >140 b/min or sustained increase/decrease >20%
- Systolic blood pressure >180 or <90 mmHg
- Agitation, sweating, anxiety, or decreased level of consciousness

Note:

*Patients experienced condition listed for at least 2 minutes will be defined as intolerance and be mechanically ventilated and rest. Reassessment would start within 24 hours.

Bosma 2016

Methods: Single center randomized controlled trial

Patients: 50 adult patients in medical-surgical ICU, under invasive mechanical ventilation for at least 36 hours under assist/control or pressure support mode.

- Mean age: 64.84 years old; male: 50%
- Mean duration of mechanical ventilation: 5.79 days
- Mean severity: APACHE II: 26.54
- Reason for intubation: Pneumonia (28%), non-respiratory sepsis (16%), cardiac arrest (14%), AECOPD (4%), post-operation (10%), CHF (2%), ARDS (4%), hepatic encephalopathy (4%)
- Comorbidities: COPD (20%), restrictive lung disease (6%), asthma (2%), diabetes (42%), CHF (6%), ischemic heart disease (10%), immunosuppression (12%), stroke (4%).

Interventions: Puritan-Bennett 840 ventilators

- Intervention: PAV+, the assist was started at 70%
- Comparison: PSV, the pressure support was started at 15 cmH_2O

The level of assist was decreased every 2-3 hours as tolerated, maintaining a respiratory rate <35 b/min and tidal volume (Vt) >5ml/kg, and pH≥7.35.

Outcomes:

- Weaning success, extubation failure was defined as the requirement of invasive ventilation within 48 hours after extubation, and of at least 12 hours of noninvasive ventilation per 24-hour period.
- Length of stay in ICU and in hospital
- ICU and hospital Mortality rate
- Reintubation rate
- Noninvasive ventilation use post-final extubation

Notes

Study protocol

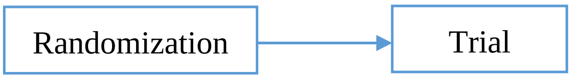

- Both protocols used a daily two-step strategy of assessing readiness to wean by calculating a "rapid shallow breathing index (RSBI)", followed by a spontaneous breathing trial (SBT).

Inclusion criteria

- Partial or complete reversal of the cause of respiratory failure
- Absence of uncontrolled, severe infection (body temperature of <36.0 ℃ or >39.0 ℃ or the presence of febrile neutropenia
- Metabolic disorders corrected: pH >7.32
- Hemoglobin >7 g/dL without ongoing bleeding
- Hemodynamic stability: requiring ≤10 μg/min norepinephrine or equivalent to support SBP>90 mmHg, no active cardiac ischemia (dynamic ST segment changes) or unstable arrhythmias (heart rate (HR) >50 and <140) or uncontrolled hypertension (SBP<180)

	● intact respiratory drive, not receiving neuromuscular blockade and able to trigger the ventilator<br>● PaO_2 >60 mmHg or a SpO_2 >90% with $FiO_2 \leq 60\%$ and PEEP ≤15cmH_2O<br>● Plateau pressure <30 cmH_2O, defined as the pressure control or pressure support level plus the PEEP equal to <30 cmH_2O<br>● For patients with ARDS, the order for low tidal volume restriction had been removed by the attending physician
Exclusion criteria	● The patient had successfully tolerated a spontaneous breathing trial on pressure support ≤ $5cmH_20$ for ≥30 minutes and was comfortably tolerating pressure support <8 cmH_2O while awaiting extubation.<br>● The patient was being considered for withdrawal of life support within the next 48 hours.<br>● The patient had a high spinal cord injury or was diagnosed with a neuromuscular or neurologic disease of a progressive nature that could result in chronic ventilator dependence and/or was a neurosurgical patient.
Failure criteria*	● Respiratory rate >35 b/min<br>● Heart rate >140 b/min or >20% increase from baseline<br>● Systolic blood pressure >180 mmHg or <90 mmHg or >30% increase from baseline<br>● Marked use of accessory muscles<br>● Abdominal paradox<br>● Diaphoresis, increased anxiety, or marked complaint of dyspnea

Note:

ARDS, acute respiratory distress syndrome.

*Respiratory distress was defined as any 2 of the criteria listed met. If respiratory distress developed at any level, the respiratory therapist increased the support to the previous level and could not decrease support for a minimum of 6 hours. If respiratory distressed continued, support could be increased to a maximum of 90% on PAV+ or 20 cmH2O on PSV.

Botha 2018

Methods: Single center randomized controlled trial

Patients: 50 adult patients in medical ICU, under invasive mechanical ventilation for at least 24 hours and ventilated with a pressure or a volume controlled mode.

- Mean age: 63.2 years old; male: 59.20%
- Mean duration of mechanical ventilation: 3.37 days
- Mean severity: APACHE II: 76.7
- Reason for intubation: respiratory diseases (20.41%), cardiac diseases (16.33%), neurological diseases (2.04%), sepsis (26.53%), gastrointestinal diseases (12.24%).
- The diagnosis of ICU admission and the comorbidities were not clearly available.

Interventions: Puritan-Bennett 840 ventilators

- Intervention: PAV+, the assist was started at 70% and weaned to 30%, decreased by 10% as tolerated according to arterial blood gases (ABG), tidal volume (tv), work of breath (WoB), respiratory rate (RR), and accessory muscle use. The support level was increased or the patient returned to mandatory mode if signs of respiratory distress were noted. Patient with continuous distress would receive up to 90% assist. The patient was deemed ready for extubation when tolerating PAV+ with 30% support, PEEP ≤5 cmH_2O, FiO_2 ≤40%, and was obeying commands. Hypoxaemia was managed by adjusting the PEEP or FiO_2.
- Comparison: PSV, Pressure support was started on the PSV level required and weaned to 10 cmH_2O as tolerated, according to ABG, tv, WoB, RR, and accessory muscle use. Pressure support level was increased or the patient returned to a mandatory mode if signs of respiratory distress were noted. The patient was deemed ready for extubation when tolerating ventilation with a PSV of 10 cmH_2O, PEEP ≤5 cmH_2O, FiO_2 ≤40%, and was obeying commands.

Outcomes:

- Weaning success: extubation failure was defined as reintubation within 48 hours.
- Duration of mechanical ventilation
- Duration of weaning
- Length of stay in ICU and in hospital
- ICU and hospital mortality
- Requirement for rescue ventilation (requiring a mandatory mode of ventilation after having commenced weaning on a spontaneous mode of ventilation), sedative drugs, or tracheostomy

Notes

Study protocol

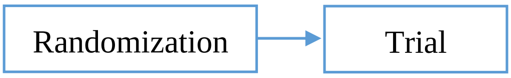

Inclusion criteria:

- Patients be anticipated to be spontaneously ventilated for at least 48 hours after randomization.
- Patients without brain death, hypoxic brain injury, tracheostomy, or neuromuscular

	disease
Exclusion criteria	Not available
Failure critieria*	● Respiratory rate >30 b/min<br>● Decreased tidal volume<br>● Increased accessory muscle use

Note:

*Patients having respiratory distress during the trial would receive increased supportive level by increasing the percentage of assist or PEEP in PAV+ group and by increasing the pressure support in PSV group. The patient would return to a mandatory mode if respiratory distress persisted after maximal support (90% assist in PAV+ group and 15 cmH_2O pressure support in PSV group)

Salama 2018

Methods	Single center randomized controlled trial
Patients	150 adult COPD patients in medical ICU, under invasive mechanical ventilation. The time and the modes of the ventilation were not available.<br>● The patients characteristics, including mean age, gender, mean duration of mechanical ventilation, severity, reasons for intubation were note available.
Interventions	The brand of the ventilators used was not available.<br>● Intervention: PAV+, the setting used for weaning was not available.<br>● Comparison: PSV, the setting used for weaning was not available.
Outcomes	● Weaning success: the definition of extubation failure was not available.<br>● Length of stay in ICU and in hospital<br>● ICU and hospital mortality<br>● Asynchrony index
Notes	
Study protocol	Randomization → Trial
Inclusion criteria	Not available
Exclusion criteria	Not available
Failure critieria*	Not available

Table S4. GARDE, Summary of findings

Summary of findings:

Proportional assist ventilation compared to Pressure support ventilation for Critically ill patients receiving endotracheal intubation

Patient or population: Critically ill patients receiving endotracheal intubation
Setting: In the intensive care unit
Intervention: Proportional assist ventilation
Comparison: Pressure support ventilation

Outcomes	Anticipated absolute effects* (95% CI)		Relative effect (95% CI)	№ of participants (studies)	Certainty of the evidence (GRADE)	Comments
	Risk with Pressure support ventilation	Risk with Proportional assist ventilation				
Weaning success	727 per 1,000	**844 per 1,000** (778 to 916)	**RR 1.16** (1.07 to 1.26)	634 (7 RCTs)	⨁⨁⨁◯ MODERATE [a]	
Reintubation	159 per 1,000	**78 per 1,000** (44 to 138)	**RR 0.49** (0.28 to 0.87)	484 (6 RCTs)	⨁⨁⨁◯ MODERATE	
Mortality	151 per 1,000	**100 per 1,000** (63 to 160)	**RR 0.66** (0.42 to 1.06)	470 (5 RCTs)	⨁⨁⨁◯ MODERATE	
ICU stay	The mean ICU stay was **0**	MD **1.58 lower** (2.68 lower to 0.47 lower)	-	276 (5 RCTs)	⨁⨁⨁◯ MODERATE [a]	
Duration of Weaning	The mean duration of Weaning was **0**	MD **0.01 lower** (1.3 lower to 1.28 higher)	-	122 (3 RCTs)	⨁⨁◯◯ LOW [a]	
Duration of Ventilation	The mean duration of Ventilation was **0** hours	MD **40.26 hours lower** (66.67 lower to 13.48 lower)	-	133 (3 RCTs)	⨁⨁◯◯ LOW [a]	

***The risk in the intervention group** (and its 95% confidence interval) is based on the assumed risk in the comparison group and the **relative effect** of the intervention (and its 95% CI).

CI: Confidence interval; **RR:** Risk ratio; **MD:** Mean difference

GRADE Working Group grades of evidence
High certainty: We are very confident that the true effect lies close to that of the estimate of the effect
Moderate certainty: We are moderately confident in the effect estimate: The true effect is likely to be close to the estimate of the effect, but there is a possibility that it is substantially different
Low certainty: Our confidence in the effect estimate is limited: The true effect may be substantially different from the estimate of the effect
Very low certainty: We have very little confidence in the effect estimate: The true effect is likely to be substantially different from the estimate of effect

Explanations

a. Most information from studies were unclear risk of allocation concealment and lack of blinding; therefore, the combined-studies risk of bias was felt to be serious.

Table S5. - Meta-regression analysis

Moderators	Variables	Study Number (N)	$RR_{interaction}$ (95% CI)	P-value
**Weaning Success**	Mean Age	6	1.005 (0.994 to 1.017)	0.3530
	Gender	6	1.003 (0.990 to 1.017)	0.6288
	Baseline duration of mechanical ventilation	4	0.979 (0.911 to 1.052)	0.5671
	Physiology score	4	1.000 (0.974 to 1.027)	0.9801
**Reintubation**	Mean Age	6	0.982 (0.917 to 1.052)	0.6107
	Gender	6	0.981 (0.928 to 1.036)	0.4850
	Baseline duration of mechanical ventilation	4	1.020 (0.549 to 1.895)	0.9498
	Physiology score	4	0.960 (0.715 to 1.289)	0.7858
**Mortality**	Mean Age	5	1.043 (0.883 to 1.232)	0.6218
	Gender	5	0.986 (0.915 to 1.063)	0.7196
	Baseline duration of mechanical ventilation	4	1.270 (0.581 to 2.775)	0.5485
	Physiology score	3	1.025 (0.899 to 1.169)	0.7095
**ICU length of stay**	Mean Age	5	0.894 (0.735 to 1.088)	0.2627
	Gender	5	1.005 (0.878 to 1.151)	0.9381
	Baseline duration of mechanical ventilation	3	1.789 (0.225 to 14.212)	0.5824
	Physiology score	3	0.499 (0.125 to 1.998)	0.3262
**Duration of weaning**	Mean Age	3	0.054 (0.000 to 15.077)	0.3091
	Gender	3	18.792 (0.032 to 11068.699)	0.3674
**Duration of weaning**	Mean Age	3	0.055 (0.000 to 18.403)	0.3278
	Gender	3	0.153 (0.003 to 8.915)	0.3658

$RR_{interaction}$ = interaction effect calculated by meta-regression, positive direction indicates that possible moderators might strengthen the outcomes in PAV compared with PSV.

P-value = The significant level was set as 0.05;

Figure S1. Assessment of risk of bias

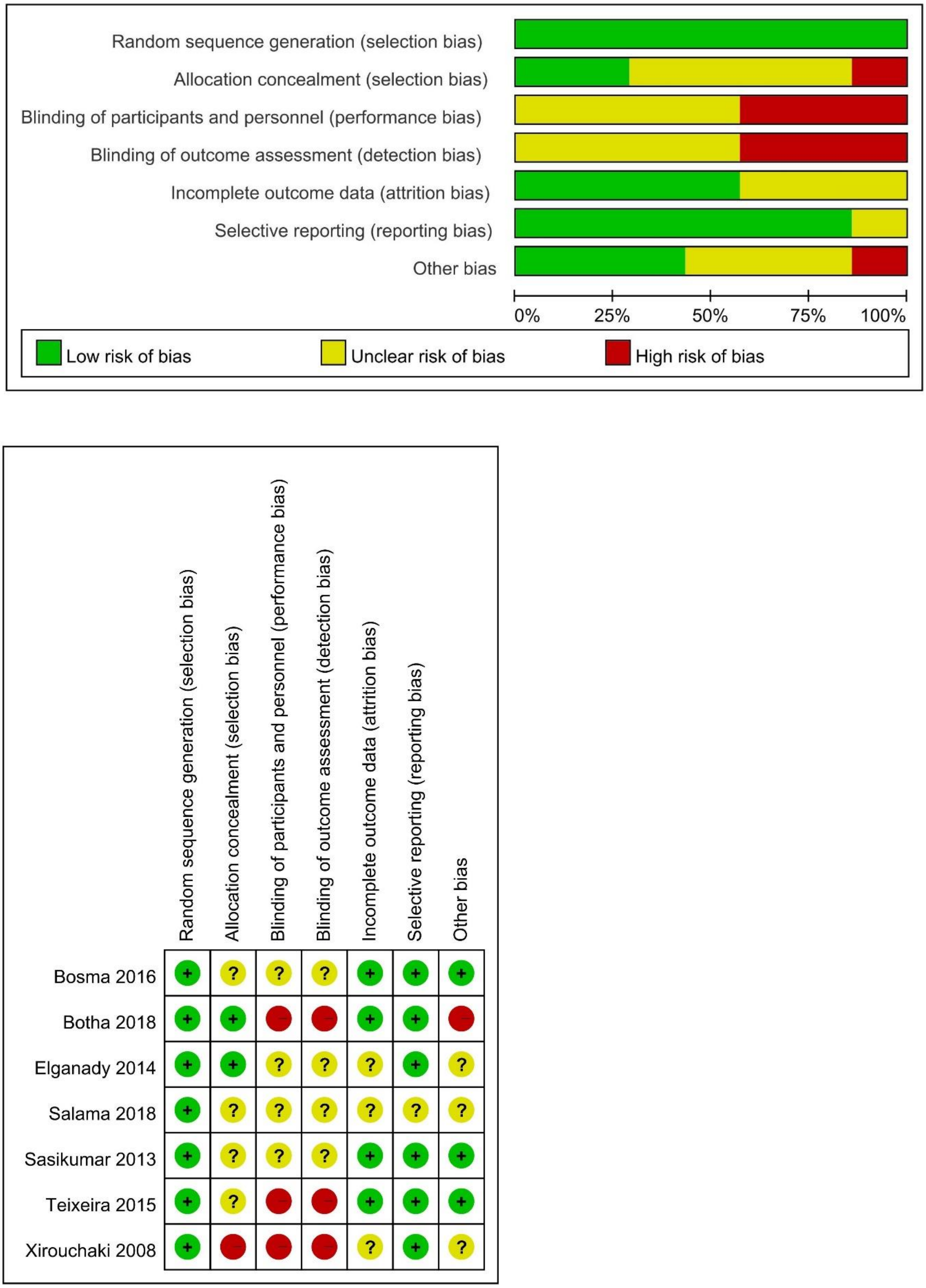

Figure S2. Subgroup analysis of outcomes

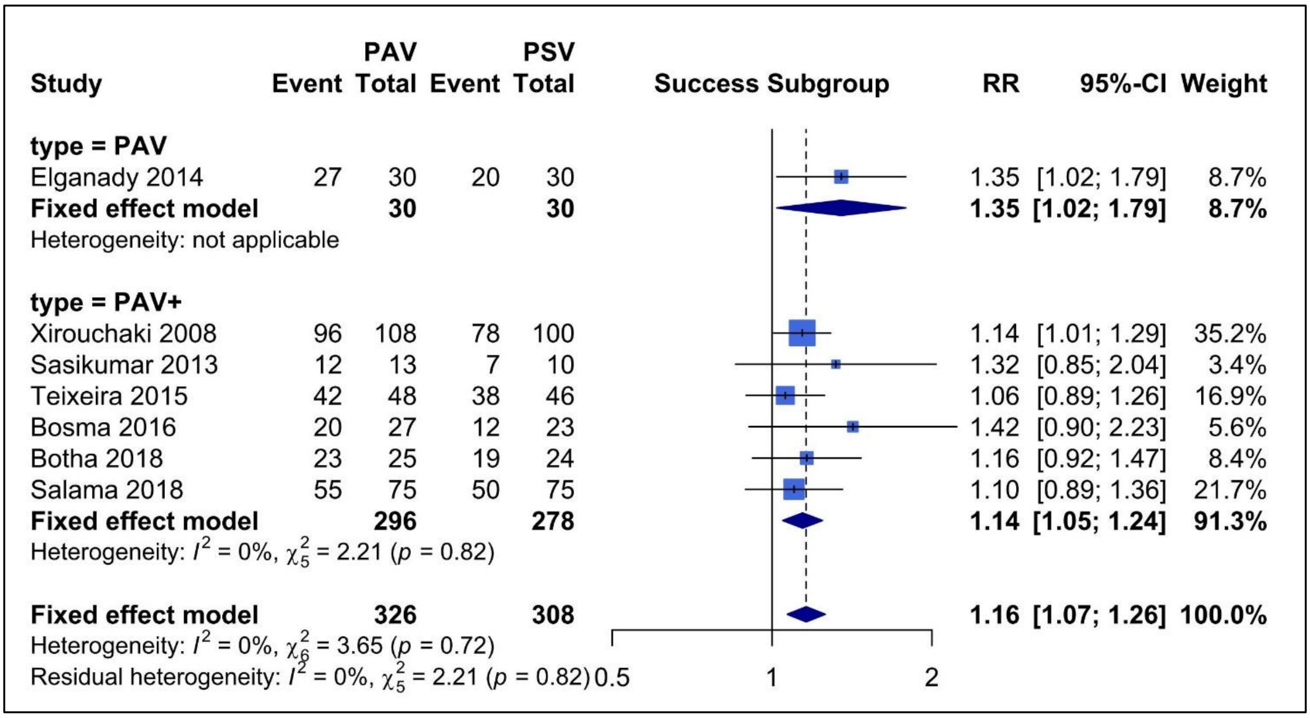

Subgroup analysis of PAV type in outcome of weaning success

The included patients were categorized by performing studies with PAV or PAV+. Outcome analyses were performed using risk ratio with related 95% confidence intervals (95%CI).

PAV, proportional assisted ventilation; PAV+, proportional assisted ventilation plus; PSV, pressure support ventilation; RR, risk ratio; CI, confidence interval

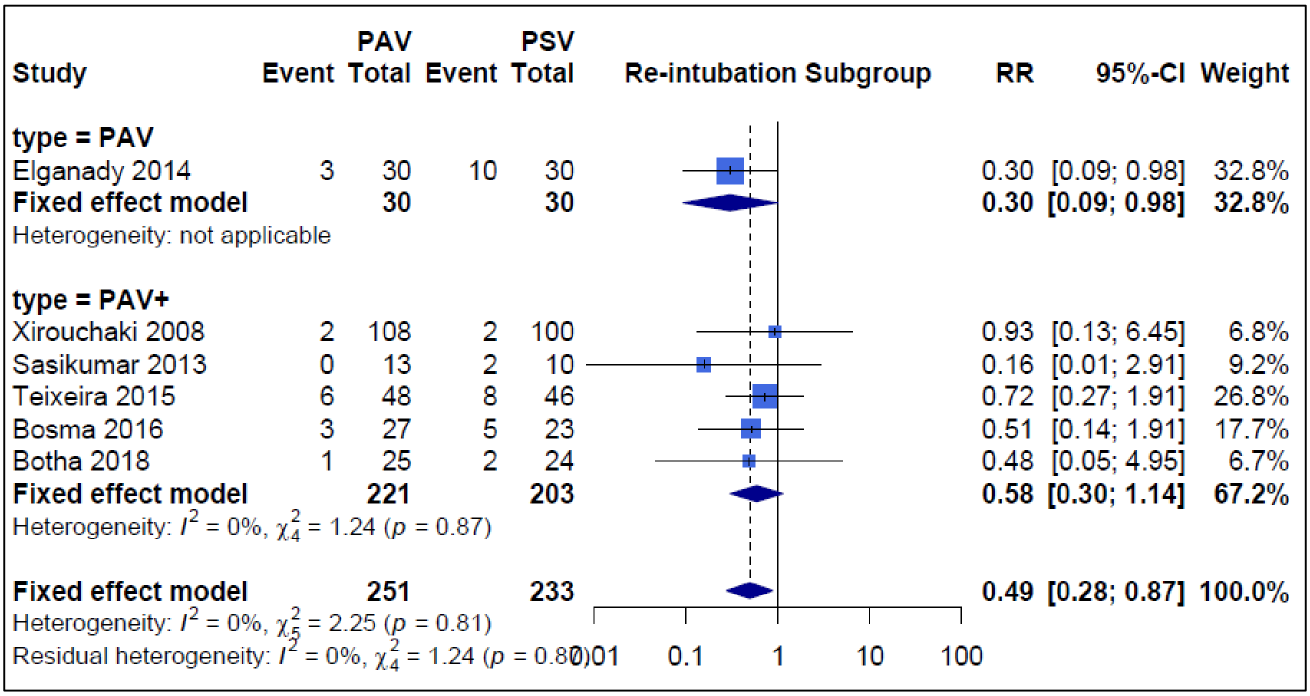

Subgroup analysis of PAV type in outcome of proportion requiring reintubation

The included patients were categorized by performing studies with PAV or PAV+. Outcome analyses were performed using risk ratio with related 95% confidence intervals (95%CI).
PAV, proportional assisted ventilation; PAV+, proportional assisted ventilation plus; PSV, pressure support ventilation; RR, risk ratio; CI, confidence interval

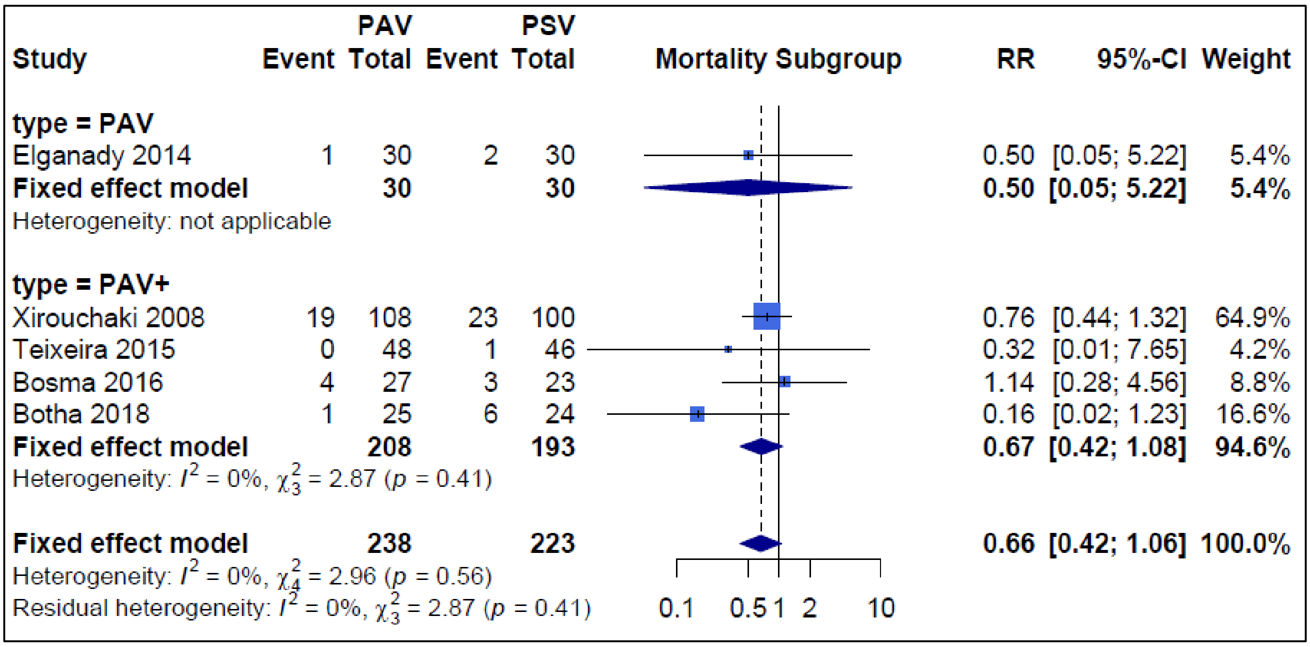

Subgroup analysis of PAV type in outcome of mortality

The included patients were categorized by performing studies with PAV or PAV+. Outcome analyses were performed using risk ratio with related 95% confidence intervals (95%CI).
PAV, proportional assisted ventilation; PAV+, proportional assisted ventilation plus; PSV, pressure support ventilation; RR, risk ratio; CI, confidence interval

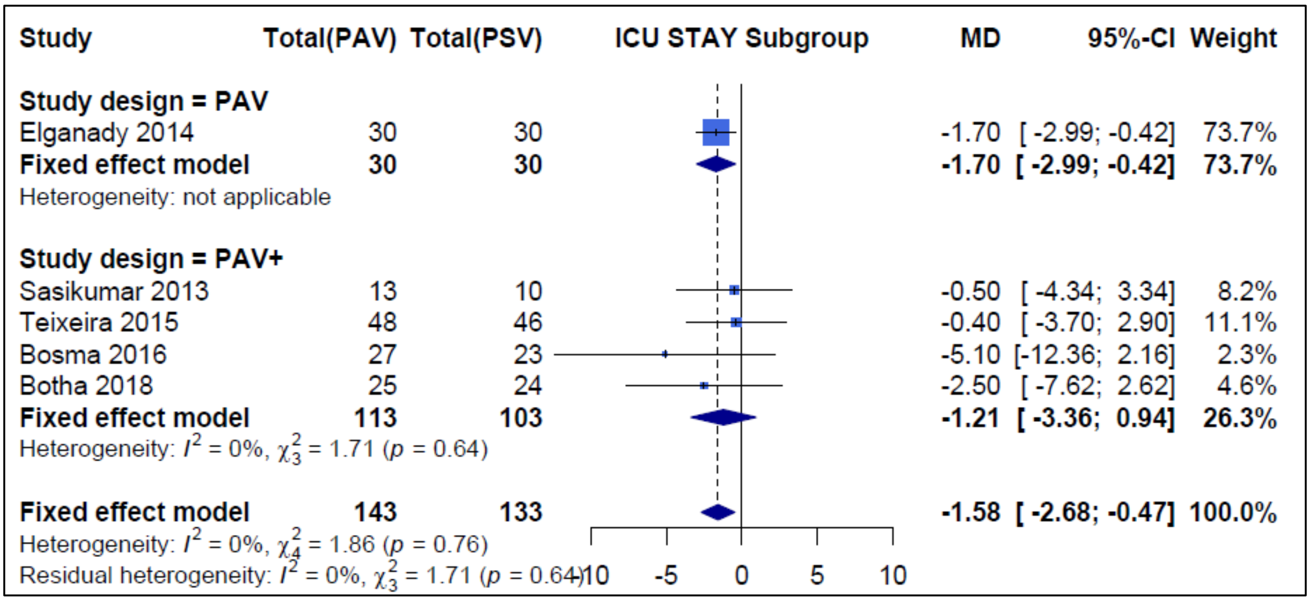

Subgroup analysis of PAV type in outcome of length of ICU stay

The included patients were categorized by performing studies with PAV or PAV+. Outcome analyses were performed using mean difference with related 95% confidence intervals (95%CI). PAV, proportional assisted ventilation; PAV+, proportional assisted ventilation plus; PSV, pressure support ventilation; MD, mean difference; CI, confidence interval

Subgroup analysis did not perform in outcomes: duration of weaning, and duration of ventilation, because there was no study involving with PAV design in those outcomes.

Figure S3. Sensitivity analysis of outcomes

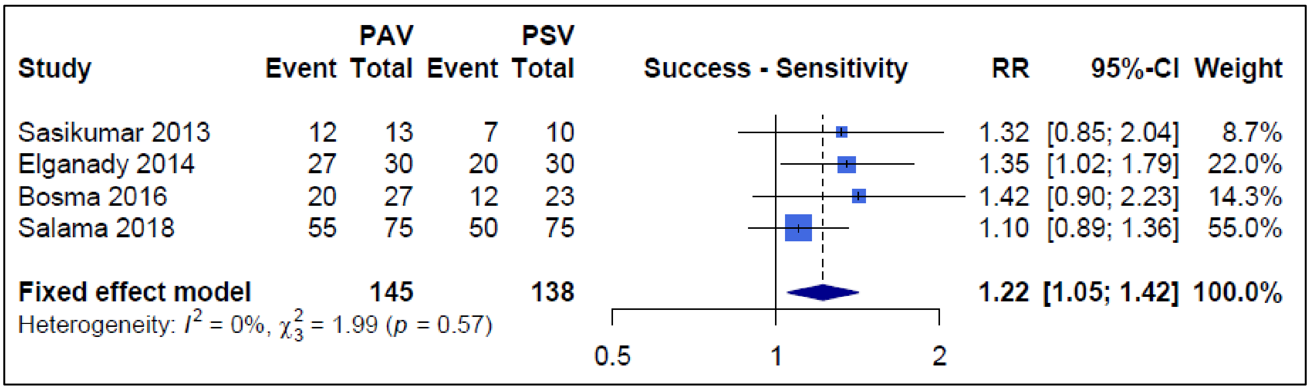

Sensitivity analysis of excluding high-risk of bias studies in outcome of weaning success

PAV, proportional assisted ventilation; PSV, pressure support ventilation; RR, risk ratio; CI, confidence interval

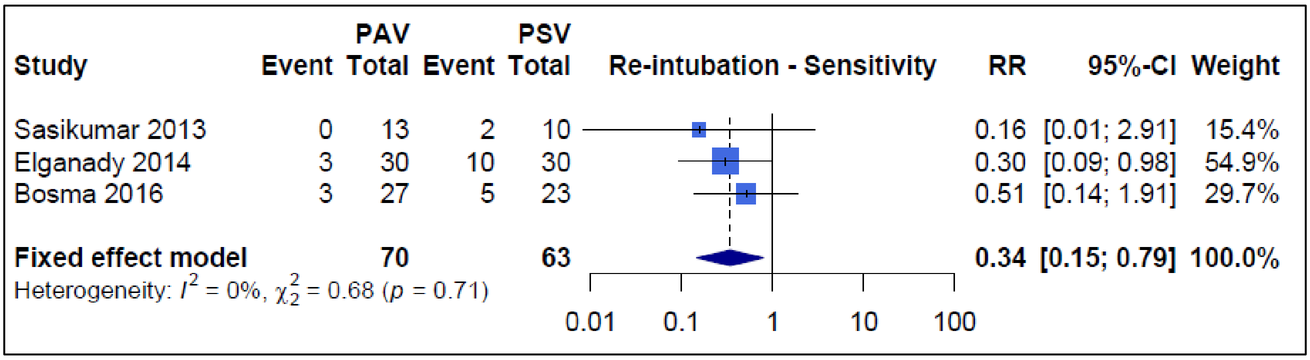

Sensitivity analysis of excluding high-risk of bias studies in outcome of proportion requiring reintubation

PAV, proportional assisted ventilation; PSV, pressure support ventilation; RR, risk ratio; CI, confidence interval

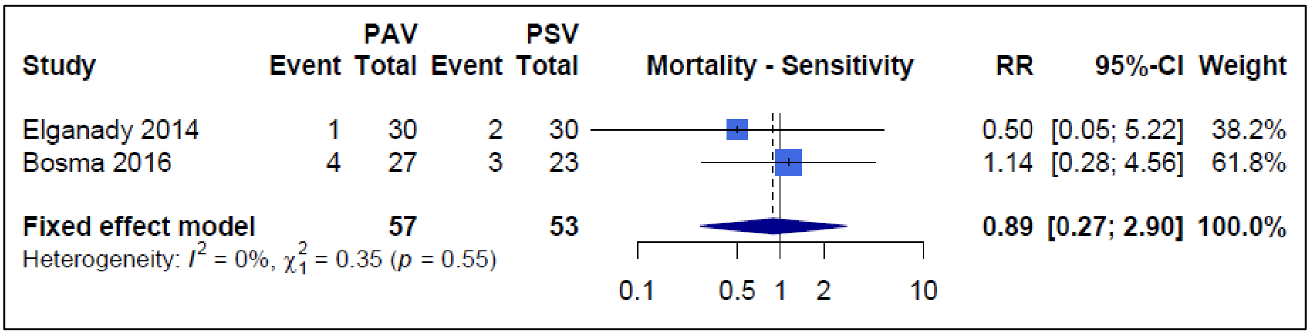

Sensitivity analysis of excluding high-risk of bias studies in outcome of mortality

PAV, proportional assisted ventilation; PSV, pressure support ventilation; RR, risk ratio; CI, confidence interval

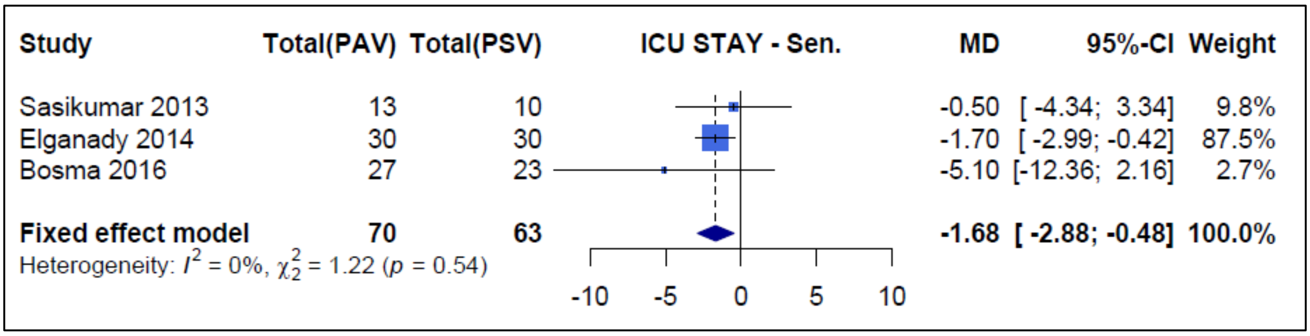

Sensitivity analysis of excluding high-risk of bias studies in outcome of ICU length of stay

PAV, proportional assisted ventilation; PSV, pressure support ventilation; MD, mean difference; CI, confidence interval

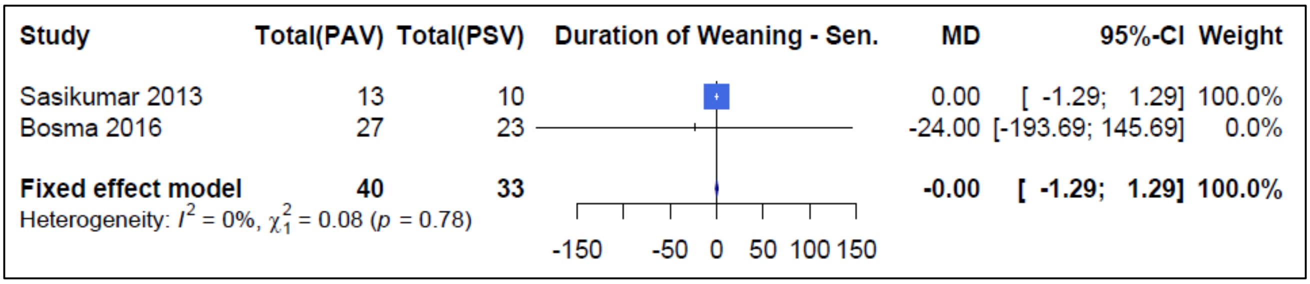

Sensitivity analysis of excluding high-risk of bias studies in outcome of duration of weaning

PAV, proportional assisted ventilation; PSV, pressure support ventilation; MD, mean difference; CI, confidence interval

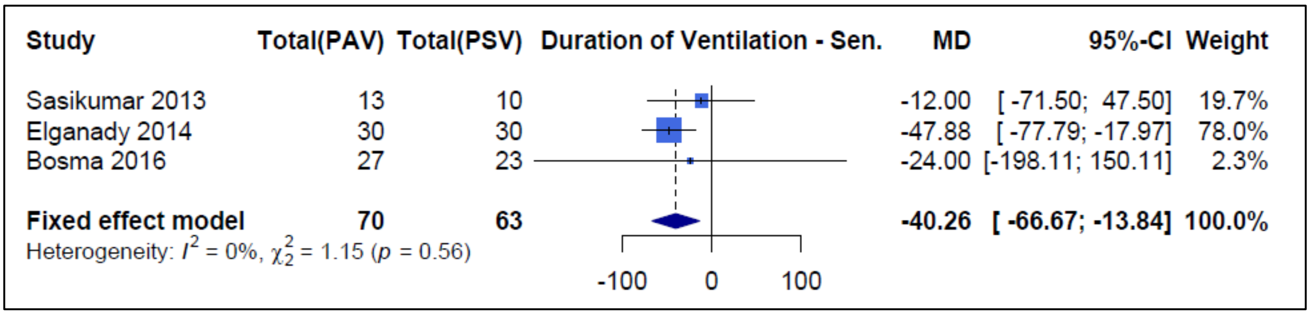

Sensitivity analysis of excluding high-risk of bias studies in outcome of duration of ventilation

PAV, proportional assisted ventilation; PSV, pressure support ventilation; MD, mean difference; CI, confidence interval

Figure S4. Funnel plots and Egger's test

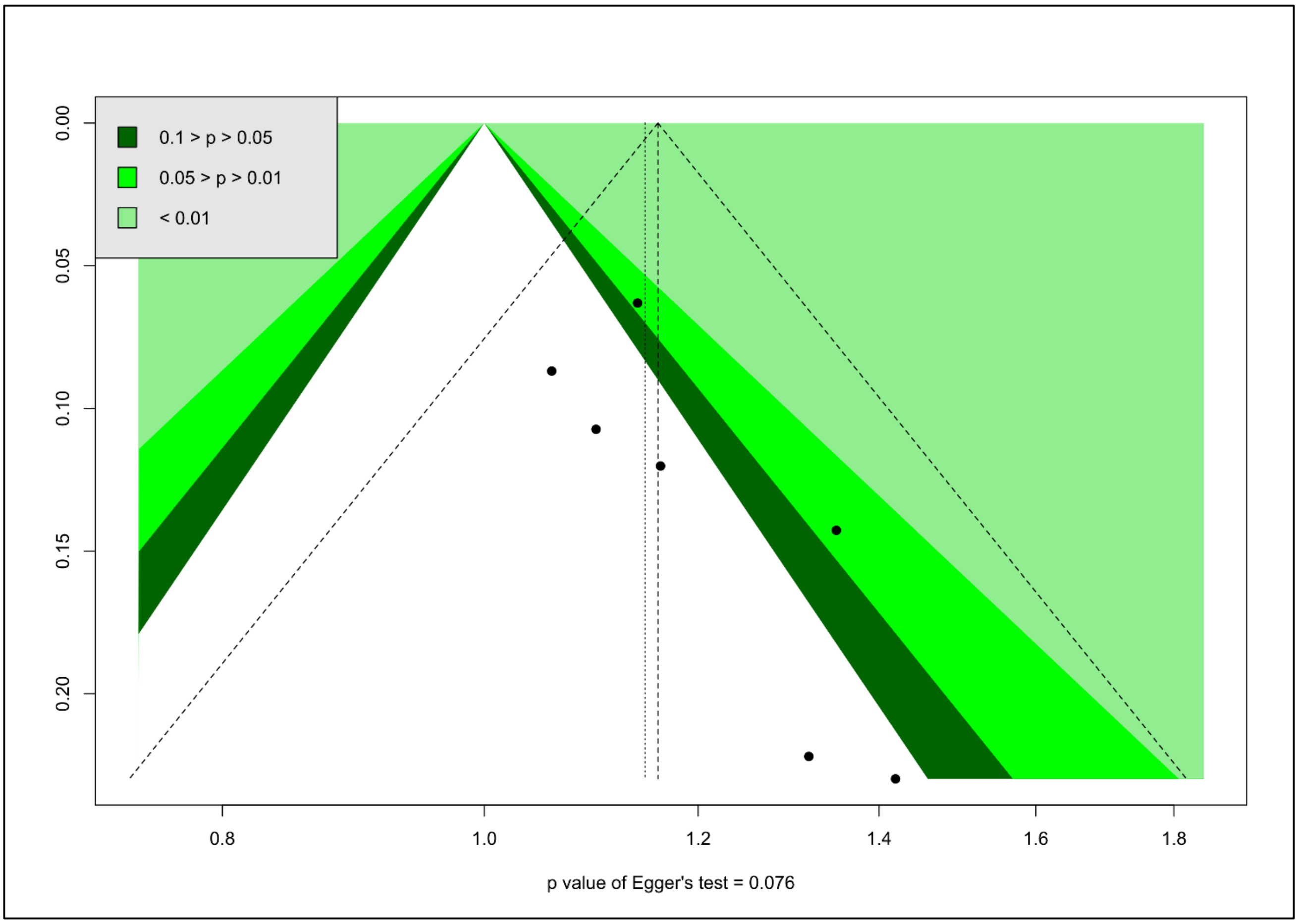

Funnel plots and Egger's test in outcome for weaning success

P-value: The significant level was set as 0.05;

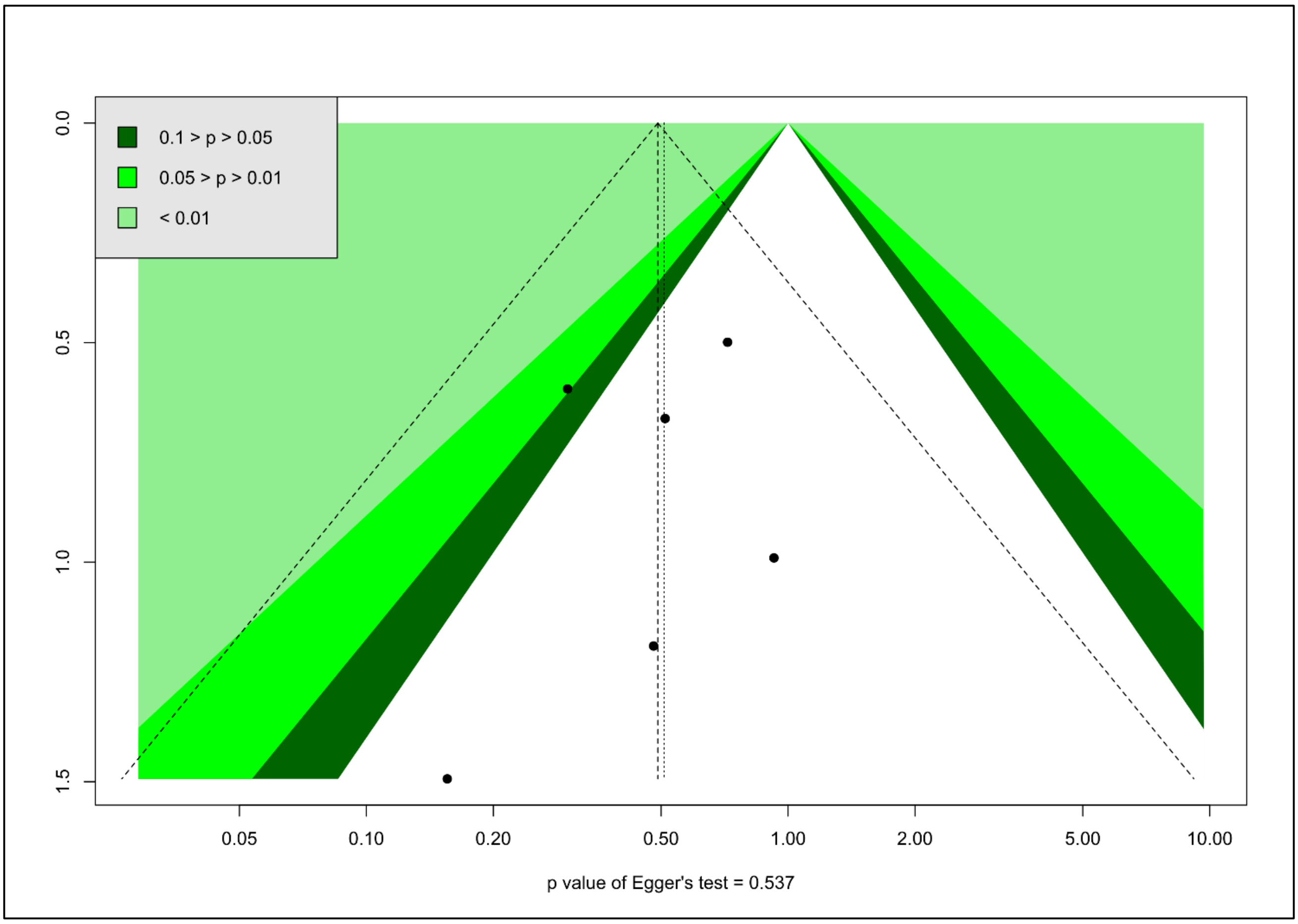

Funnel plots and Egger's test in outcome for re-intubation

P-value: The significant level was set as 0.05;

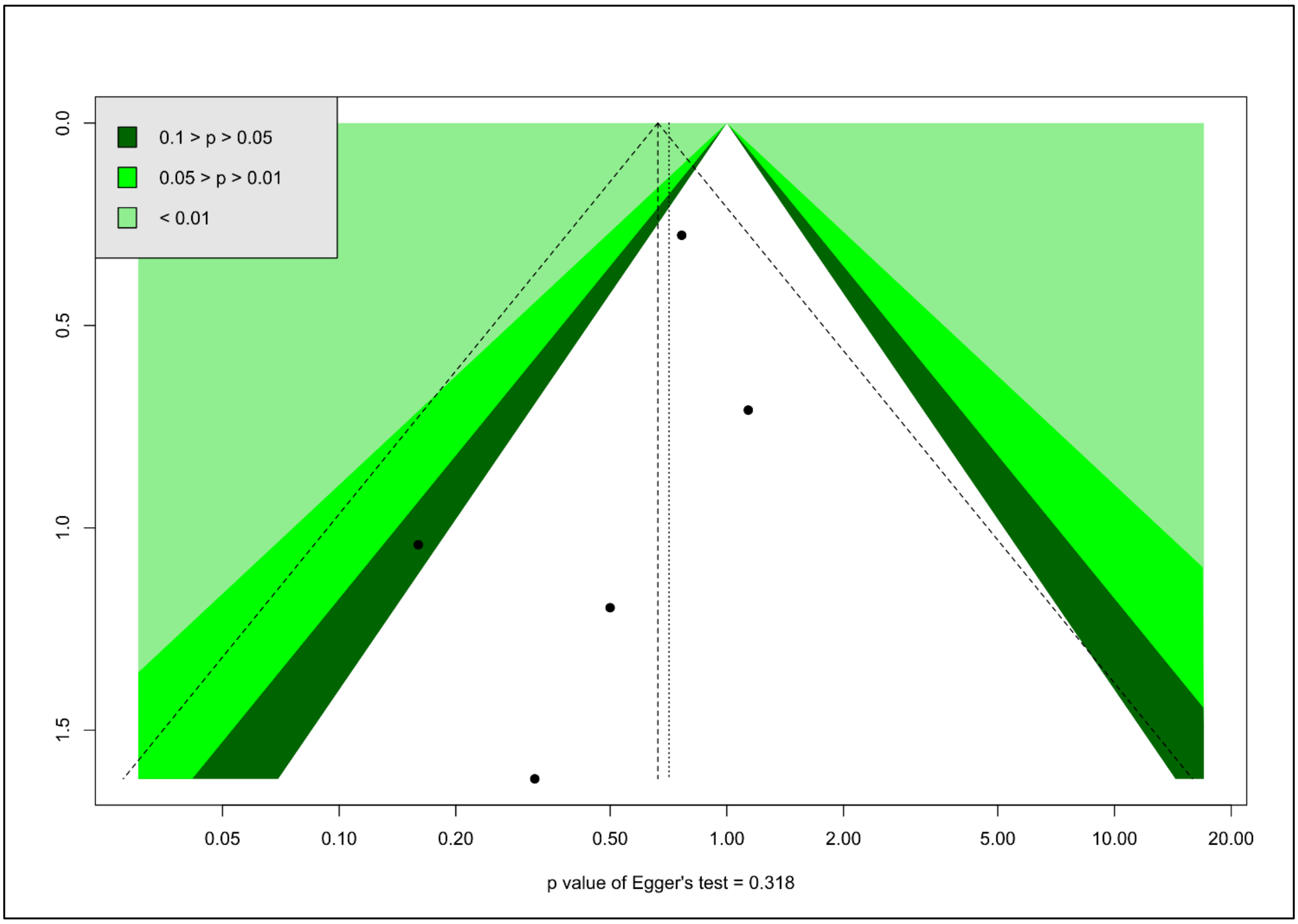

Funnel plots and Egger’s test in outcome for mortality

P-value: The significant level was set as 0.05;

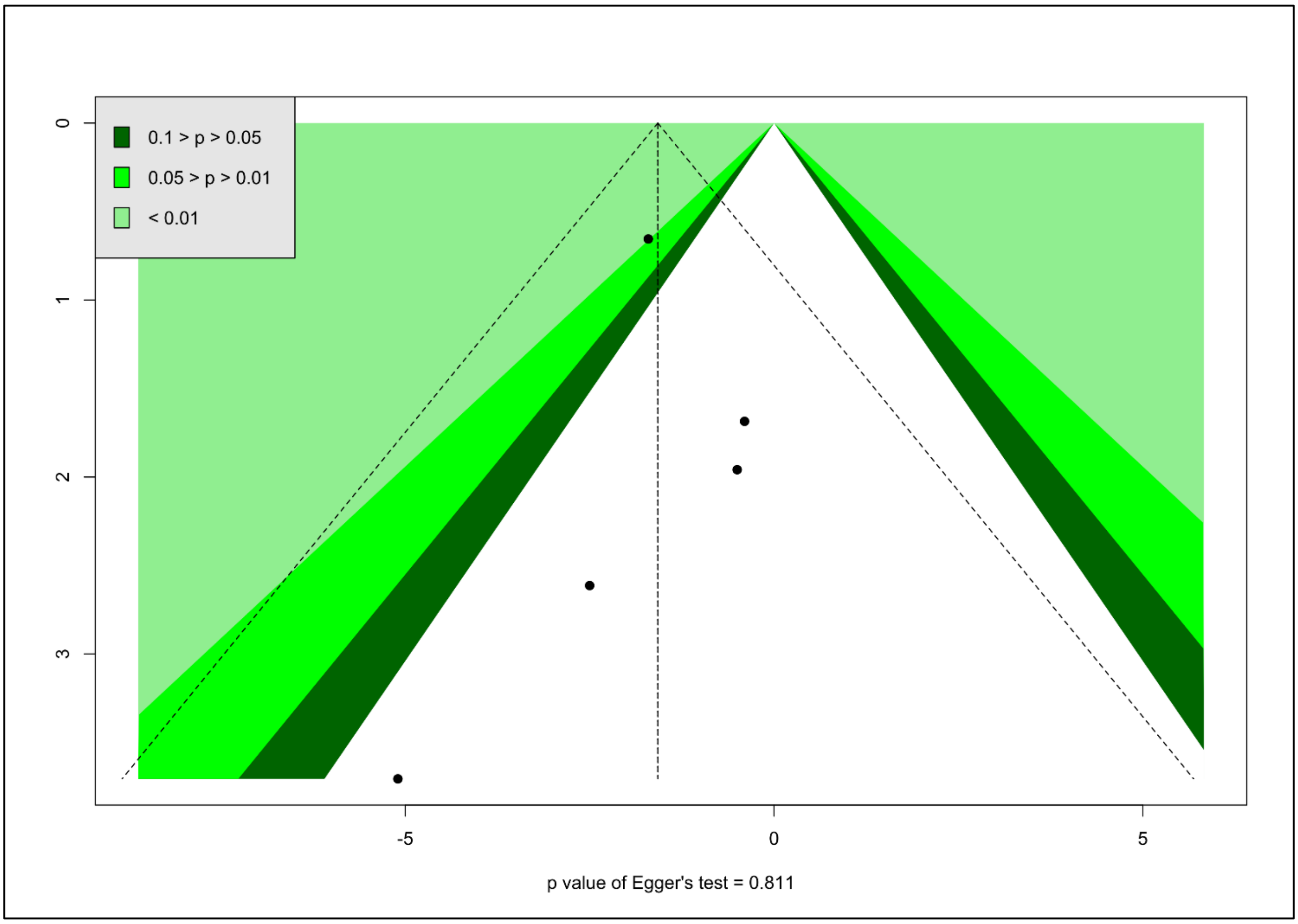

Funnel plots and Egger’s test in outcome for ICU length of stay

P-value: The significant level was set as 0.05;

Note:

The funnel plots and Egger’s test did not perform in outcome for duration of weaning and duration of ventilation, because the studies included in those outcomes were too few to conducted further examination.

Figure S5. Trial sequential analysis of secondary outcomes

The x-axis represents the accrued information size of patients and the required information size. The y axis represents the z values, representing the accumulating statistical information. The blue line (z-curve) represents the cumulative Z-value, and each square represents an individual trial. The small red lines at the top and bottom left-hand corners, trial sequential boundaries for benefit or harm, represent the threshold for statistical significance in TSA. The horizontal dark red lines represent the threshold for significance in conventional meta-analysis, at 1.96 of Z-value, corresponding of 0.05 of p-value. The red line of triangle shape represents the futility boundaries and futility area in TSA.

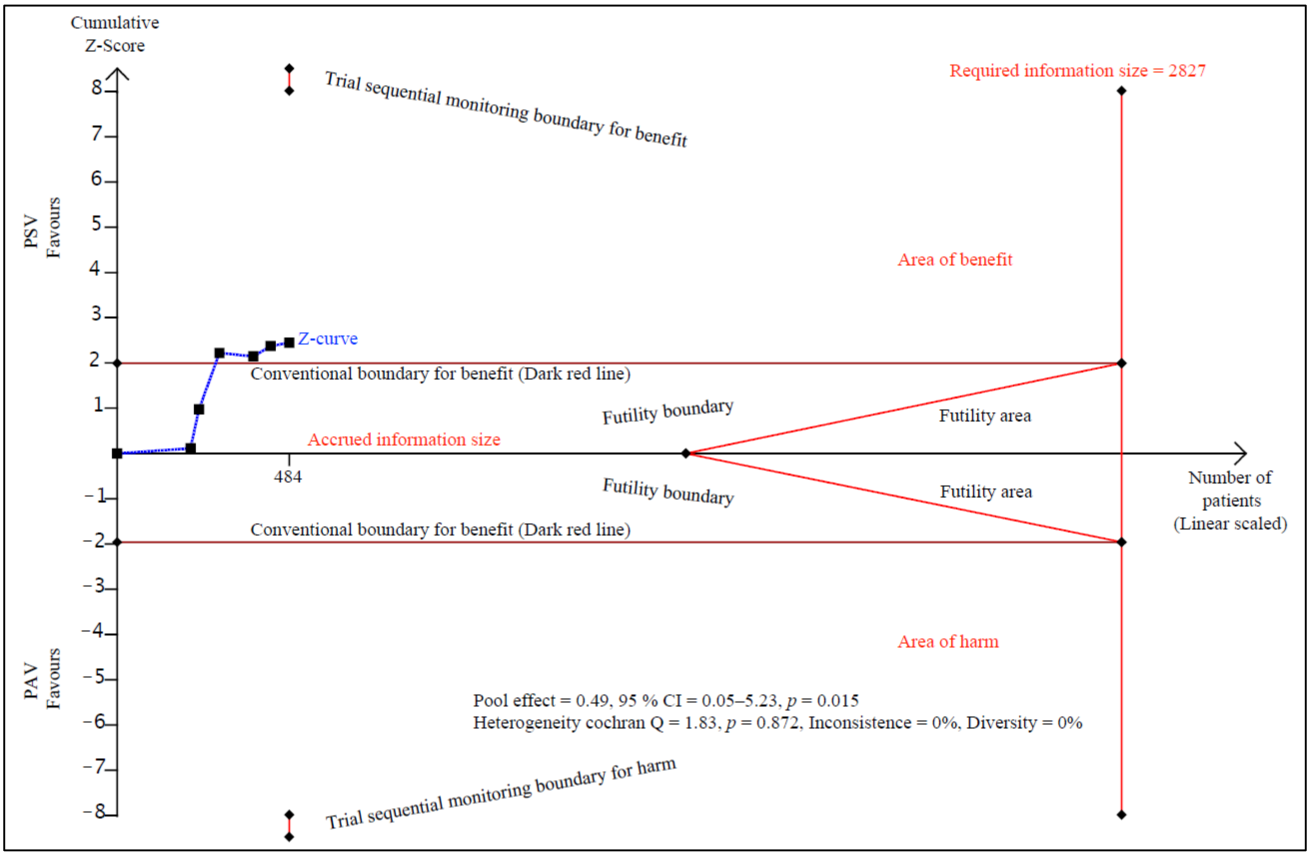

Trial sequential analysis of proportion requiring reintubation.

Trial sequential analysis of six trials reporting proportion requiring reintubation, control event proportion of 20.4%, diversity of 26%, type 1 error of 5% (α=0.05, two sided), power of 80% (β=0.20), and relative risk reduction of 20%. The required information size of 3812 has not been reached and none of the boundaries for benefit, harm or futility has been crossed, leaving the meta-analysis inclonclusive. The trial sequential analysis adjusted 95% confidence interval for an odds ratio of 0.37 is 0.02—5.71.

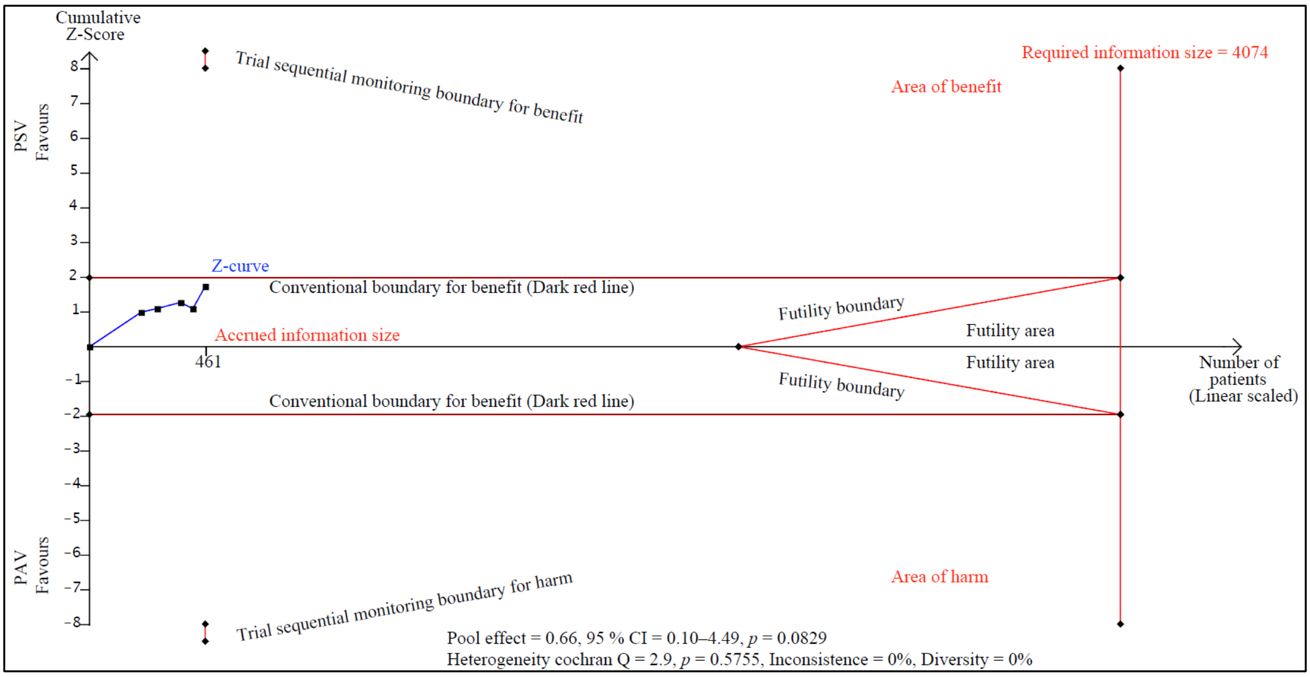

Trial sequential analysis of mortality.

Trial sequential analysis of five trials reporting mortality, control event proportion of 15%, heterogeneity correction of model variance based, type 1 error of 5% (α=0.05, two sided), power of 80% (β=0.20), and relative risk reduction of 20%. The required information size of 4074 has not been reached and none of the boundaries for benefit, harm or futility has been crossed, leaving the meta-analysis inclonclusive. The trial sequential analysis adjusted 95% confidence interval for an odds ratio of 0.72 is 0.10—5.13.

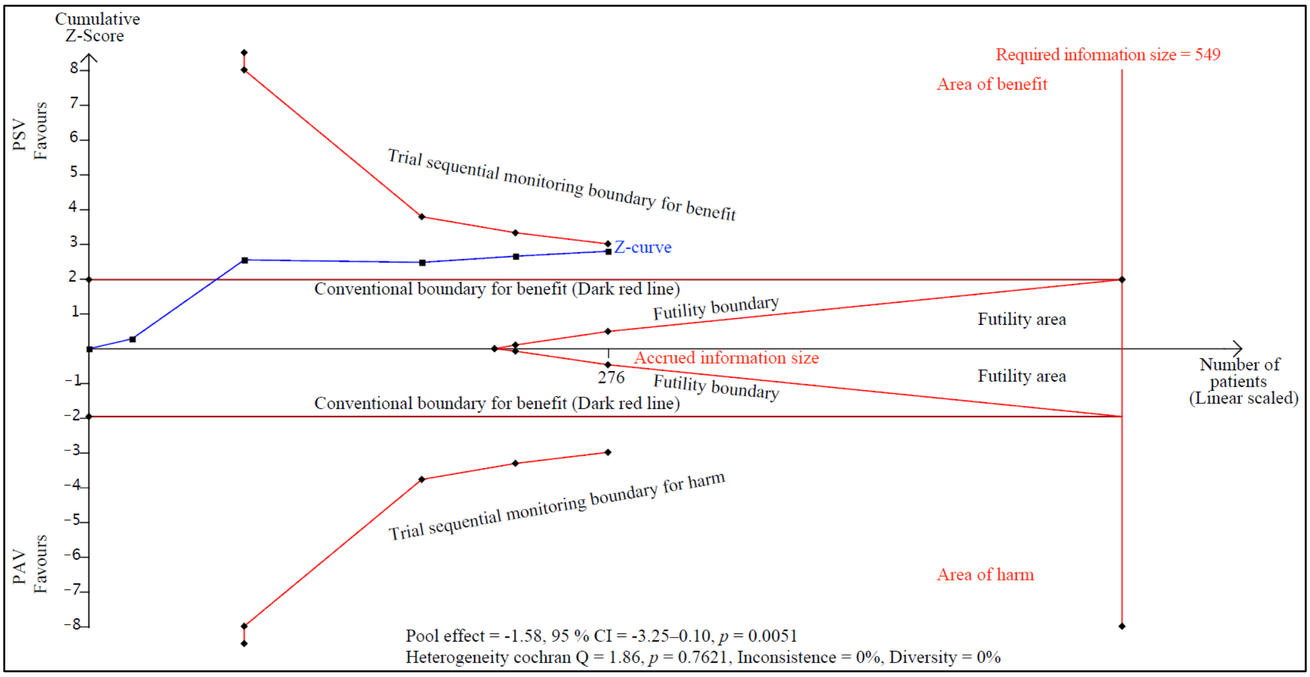

Trial sequential analysis of ICU length of stay

Trial sequential analysis of five trials reporting mortality, mean difference and variance of low-bias based, heterogeneity correction of model variance based, heterogeneity correction of model variance based, type 1 error of 5% (α=0.05, two sided), power of 80% (β=0.20). The required information size of 549 has not been reached and none of the boundaries for benefit, harm or futility has been crossed, leaving the meta-analysis inclonclusive. The trial sequential analysis adjusted 95% confidence interval for an odds ratio of -1.58 is -3.25—0.10.

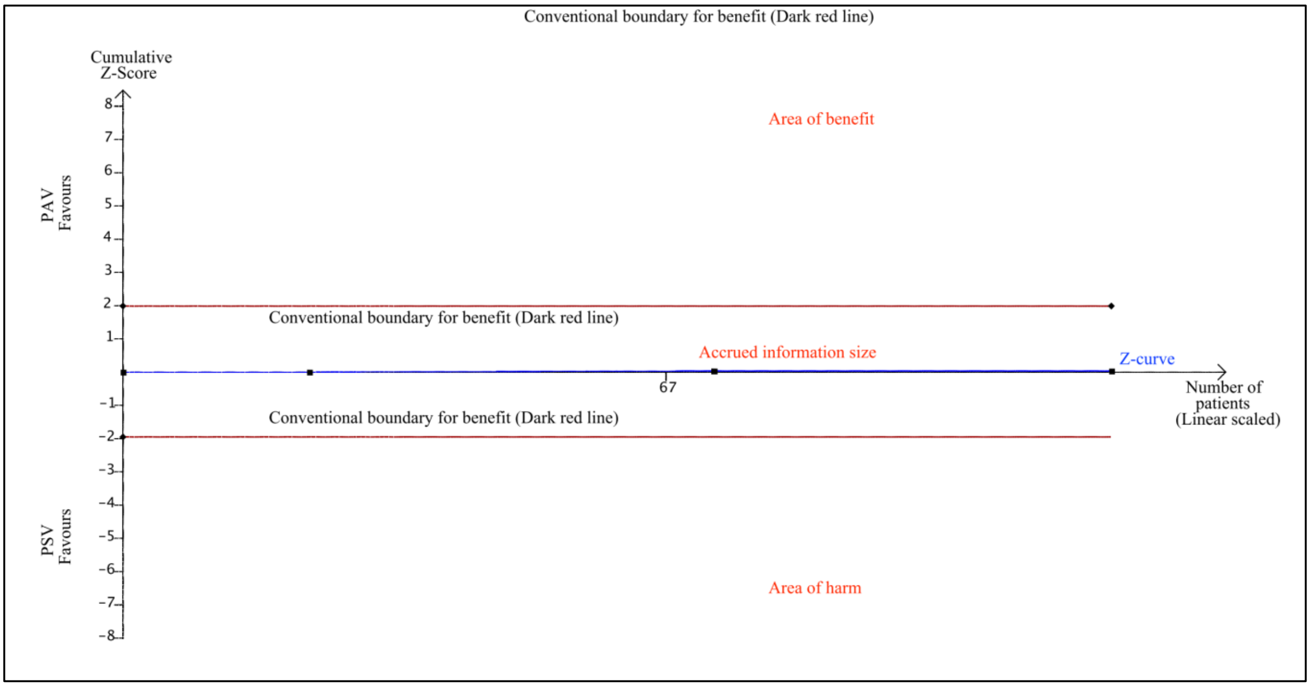

Trial sequential analysis of duration of weaning.

Trial sequential analysis of five trials reporting duration of weaning, mean difference and variance of low-bias based, heterogeneity correction of model variance based, diversity of 0%, type 1 error of 5% (α=0.05, two sided), power of 80% (β=0.20). In this case, the mean difference was too small to construct any trial sequential boundaries. Consequently, the trial sequen tial analysis could not be visualised on a graph and adjusted confidence intervals could not be calculated.

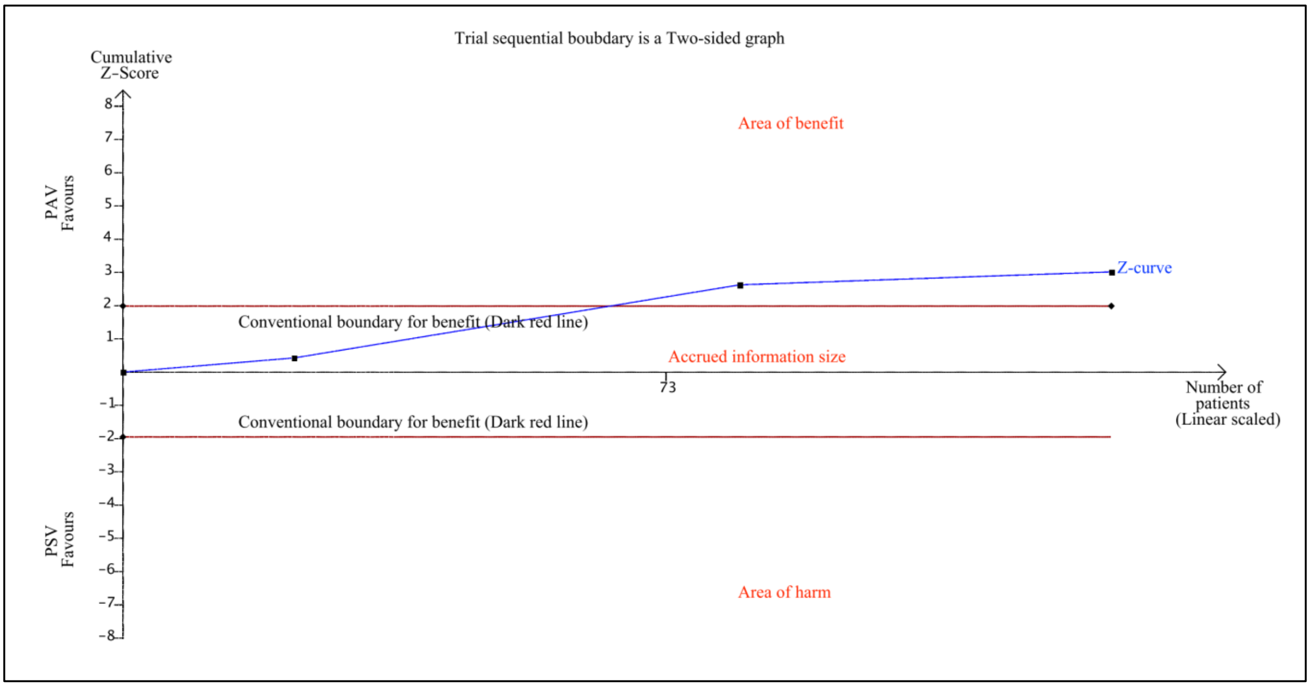

Trial sequential analysis of duration of ventilation.

Trial sequential analysis of five trials reporting duration of ventilation, mean difference and variance of low-bias based, heterogeneity correction of model variance based, diversity of 0%, type 1 error of 5% (α=0.05, two sided), power of 80% (β=0.20). In this trial sequential analysis, the data were in fact too sparse to construct futility boundaries, so these are not seen on this graph.
